# Combined MEK/MDM2 inhibition demonstrates antitumor efficacy in *TP53* wild-type thyroid and colorectal cancers with MAPK alterations

**DOI:** 10.1038/s41598-022-05193-z

**Published:** 2022-01-24

**Authors:** Seyed Pairawan, Argun Akcakanat, Scott Kopetz, Coya Tapia, Xiaofeng Zheng, Huiqin Chen, Min Jin Ha, Yasmeen Rizvi, Vijaykumar Holla, Jing Wang, Kurt W. Evans, Ming Zhao, Naifa Busaidy, Bingliang Fang, Jack A. Roth, Ecaterina Ileana Dumbrava, Funda Meric-Bernstam

**Affiliations:** 1 Department of Surgical Oncology, The University of Texas MD Anderson Cancer Center, Houston, TX 77030 USA; 2 Department of Investigational Cancer Therapeutics, The University of Texas MD Anderson Cancer Center, Houston, TX 77030 USA; 3 Department of Gastrointestinal Medical Oncology, The University of Texas MD Anderson Cancer Center, Houston, TX 77030 USA; 4 Department of Translational Molecular Pathology, The University of Texas MD Anderson Cancer Center, Houston, TX 77030 USA; 5 Department of Bioinformatics and Computational Biology, The University of Texas MD Anderson Cancer Center, Houston, TX 77030 USA; 6 Sheikh Khalifa Bin Zayed Al Nahyan Institute for Personalized Cancer Therapy, The University of Texas MD Anderson Cancer Center, Houston, TX 77030 USA; 7 Department of Endocrine Neoplasia and Hormonal Disorders, The University of Texas MD Anderson Cancer Center, Houston, TX 77030 USA; 8 Department of Thoracic and Cardiovascular Surgery, The University of Texas MD Anderson Cancer Center, Houston, TX 77030 USA; 9 Department of Breast Surgical Oncology, The University of Texas MD Anderson Cancer Center, 1400 Holcombe Blvd, FC8.3044, Houston, TX 77030 USA; 10 Present Address: Epizyme Inc., Boston, USA

**Keywords:** Thyroid cancer, Colorectal cancer, Targeted therapies

## Abstract

Most tumors with activating MAPK (mitogen-activated protein kinase) pathway alterations respond poorly to MEK inhibitors alone. Here, we evaluated combination therapy with MEK inhibitor selumetinib and MDM2 inhibitor KRT-232 in *TP53* wild-type and MAPK altered colon and thyroid cancer models. In vitro, we showed synergy between selumetinib and KRT-232 on cell proliferation and colony formation assays. Immunoblotting confirmed p53 upregulation and MEK pathway inhibition. The combination was tested in vivo in seven patient-derived xenograft (PDX) models (five colorectal carcinoma and two papillary thyroid carcinoma models) with different *KRAS*, *BRAF*, and *NRAS* mutations. Combination therapy significantly prolonged event-free survival compared with monotherapy in six of seven models tested. Reverse-phase protein arrays and immunohistochemistry, respectively, demonstrated upregulation of the p53 pathway and in two models cleaved caspase 3 with combination therapy. In summary, combined inhibition of MEK and MDM2 upregulated p53 expression, inhibited MAPK signaling and demonstrated greater antitumor efficacy than single drug therapy in both in vitro and in vivo settings. These findings support further clinical testing of the MEK/MDM2 inhibitor combination in tumors of epithelial origin with MAPK pathway alterations.

## Introduction

The MAPK (mitogen-activated protein kinase) pathway is one of the most important and commonly altered signaling cascades in cancer^1,2^. Mutations affecting the RAS protein isoforms have been found in 17% of human cancers, with activating mutations in Kirsten rat sarcoma viral oncogene homolog (*KRAS*) being the most common (84%)^3^. *KRAS* mutations (hot spot missense mutations in exon 2 codon 12, 13, and 61) have been observed in multiple tumor types, including pancreatic (> 90%), colon (45%), and lung cancers (35%)^4^. Mutations in the other two RAS isoforms (*NRAS* and *HRAS*) represent 12% and 4%, respectively, of all RAS mutations^3^. Presence of a *KRAS* mutation has been reported as a negative predictive factor in non-small cell lung cancer and is known to limit the efficacy of targeted therapy directed against epidermal growth factor receptor (EGFR) in colorectal cancer (CRC)^5^. *BRAF* mutations can also activate the MAPK pathway. The most common *BRAF* mutation is the BRAF V600E missense mutation^6^, which is present in 60% to 70% of patients with papillary thyroid cancer (PTC), 60% of those with melanoma, and 18% of those with CRC^7^. The presence of *BRAF* mutations has been associated with more advanced cancer stage at diagnosis and higher risk of relapse^7,8^.

Tumor suppressor protein p53 (encoded by the *TP53* gene) inhibits the development of malignancy through modulating apoptosis and cell cycle arrest^9^. *TP53* is the most commonly altered gene in human cancers^10^. Loss or decreased activity of its wild-type form occurs through inactivation of *TP53* by mutations and/or deletions of the gene or through amplification and/or overexpression of its negative regulators such as murine double minute 2 (MDM2) and murine double minute X (MDMX)^11,12^. MDM2 negatively regulates p53 through direct binding of the N-terminal of p53 and via ubiquitin-mediated transport for proteasomal degradation through the E3-ligase activity of MDM2^13^. The p53 signaling pathway in tumors with wild-type *TP53* (*TP53 WT*) is commonly affected by amplification or overexpression of *MDM2*, and this was demonstrated to be a major driver across malignancies with different histological characteristics, including breast cancer, colorectal cancer, lung cancer, sarcoma, and melanoma^13,14^. One approach to suppress tumor growth involves targeting MDM2-p53 interaction in tumors with *TP53 WT* and amplified *MDM2* gene by using MDM2 inhibitors in order to restore p53 activity and induce apoptosis and cell cycle arrest^15^. Preclinical and clinical studies have demonstrated promising results in which MDM2 inhibitors induced growth inhibition or tumor regression^16,17^.

KRT-232 is an oral small molecule inhibitor of MDM2-p53 interaction^18^. KRT-232 binds to the p53 binding pocket of MDM2, thereby prevents MDM2 from binding to p53^18^. There are several preclinical studies investigating therapeutic potential of KRT-232^18–20^. KRT-232 monotherapy showed significantly suppressed tumor growth and complete tumor regression in 10 of 12 sarcoma *TP53 WT* xenograft models^18^. MEK inhibitors are approved by the US Food and Drug Administration for use in combination with BRAF inhibitors for the treatment of metastatic melanoma harboring BRAF V600E/K mutation. These MEK inhibitors have similar but not identical activity mechanisms^21^. However, currently MEK inhibitors are not approved for treatment of *KRAS* mutations. In preclinical lung cancer models, MEK inhibitors, which target downstream of RAS and RAF, demonstrated effectiveness in models with KRAS G12C mutation and increased sensitivity to chemotherapeutic agents^22^. Subsequent human clinical trials failed to show any improvement in response rate^23^. Selumetinib is a selective ATP non-competitive inhibitor of MEK1/2 currently being explored in clinical trials^24^ and was recently approved by the Food and Drug Administration for pediatric neurofibromatosis type 1 patients with inoperable plexiform neurofibromas^25^. Recent efforts in developing selective KRAS G12C inhibitors have demonstrated promising activity in preclinical and clinical studies^26–28^, but effective strategies for other *KRAS* mutations are lacking. Combinatorial attempts have been made, including a study of a MEK inhibitor plus an AKT inhibitor, which demonstrated promising preclinical data; however, these findings failed to translate to the clinic, at least in part because overlapping toxicities limited the ability to achieve exposures needed for clinical activity^29^.

Saiki et al. screened a panel of cell lines and showed a strong and widespread synergy between MDM2 and MEK inhibitors, independent of MAPK pathway mutations^30^. Berberich et al. showed that one mechanism of resistance to MDM2 inhibition is the activation of the MAPK pathway in glioblastoma and that the combination of MEK and MDM2 inhibitors overcame this^31^. In dedifferentiated liposarcoma cell lines and patient-derived xenograft (PDX) models, MDM2 inhibitor monotherapy upregulated phosphorylation of extracellular signal-regulated kinase (p-ERK) and activated the MEK/ERK signaling pathway^32^. Combining MDM2 and MEK inhibitors decreased p-ERK and had significant antitumor synergy^32^. However, it remains unknown if this combination therapy is effective across multiple tumor types (such as PTC and CRC), or with different genomic alterations activating MAPK signaling. We therefore sought to evaluate the combined antitumor efficacy of MDM2 inhibition (KRT-232) and MEK inhibition (selumetinib) in both CRC and PTC with MAPK alterations and with *TP53 WT* in both cell lines and PDXs. We found that the combination of KRT-232 with selumetinib demonstrated synergistic activity and superior antitumor efficacy compared to either single agent alone in both in vitro and in vivo PTC and CRC models.

## Results

### Selumetinib combined with KRT-232 demonstrates synergistic in vitro antiproliferative activity

**Figure 1:**
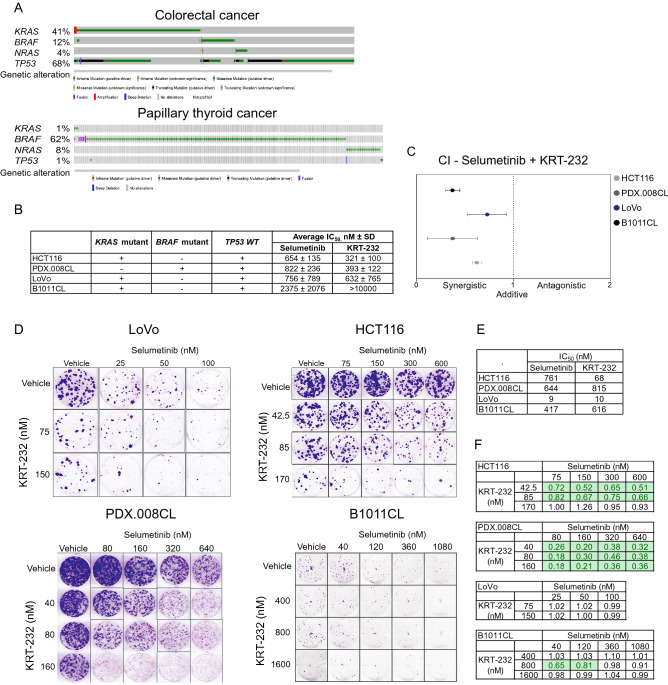
In vitro synergistic effects of MDM2 and MEK inhibition in CRC and PTC. **(A)** *TP53* and MAPK pathway gene alteration frequencies in human CRCs and PTCs. **(B**,**C)** Three CRC cell lines (LoVo, HCT116, and B1011CL) and one PTC cell line (PDX.008CL) were treated for 72 h with increasing concentrations of vehicle, selumetinib, KRT-232, or a combination of both. Drug toxicity and cell growth were determined using sulforhodamine B assay. Individual IC_50_ values **(B)** and combination indices **(C)** are shown. KRT-232 IC_50_ was beyond the tested drug concentration range. CI < 1 indicates synergistic activity. IC_50_ and CI values were calculated using CalcuSyn. **(D)** Colony formation assays of three CRC and one PTC cell line. Cells were treated with vehicle or various doses of selumetinib, KRT-232, or their combination for 2–3 weeks. This was followed by fixation of colonies and staining using crystal violet. **(E)** Individual IC_50_ values were calculated using CalcuSyn. **(F)** The Bliss independence model was used to evaluate the drug interaction across all dose combinations tested. Interaction index values < 1 indicate synergy and values within 0–0.9 range were highlighted in green. All experiments were repeated at least three times.

Many CRC and PTC patients with MAPK pathway alterations have *TP53 WT* status. To determine the clinical feasibility of combined MDM2 and MEK inhibition in colorectal and thyroid cancers, we assessed the *TP53 WT* status and the presence of MAPK alterations (i.e., *KRAS*, *BRAF*, *NRAS*) using the cBioportal database (Source of data: The Cancer Genome Atlas, Memorial Sloan Kettering Cancer Center, Genentech, Clinical Proteomic Tumor Analysis Consortium 2.0, Case Comprehensive Cancer Center for CRC and The Cancer Genome Atlas for PTC)^33^. This revealed *KRAS* missense mutation rates of 1% in PTC and 41% in CRC; *BRAF*, 62% in PTC and 12% in CRC (missense or fusion); and *NRAS*, 8% in PTC and 4% in CRC (missense) (Fig. 1A). Among patients with MAPK alterations, 32% of CRC patients and 99% of PTC patients had *TP53 WT* tumors.

To determine the combined growth inhibitory effect of selumetinib (MEK inhibitor) and KRT-232 (MDM2 inhibitor), we performed sulforhodamine B (SRB) assay in *KRAS*-mutated CRC cell lines HCT116 (KRAS G13D), LoVo (KRAS G13D), and B1011CL (KRAS A148T) and *BRAF-*mutated PTC cell line MDA-PDX.008CL (PDX.008CL, BRAF K601N). B1011CL and PDX.008CL cell lines were derived from B1011 and PDX.008 PDX models, respectively. SRB binds to proteins, and the amount of bound dye is used to determine the cell density and measure cell proliferation. Single-agent activity of selumetinib and KRT-232 demonstrated half maximal inhibitory concentration (IC_50_) values < 1 µM in most cell lines, which is below clinically achievable plasma concentrations in patients^34,35^, leading to significant growth inhibition (Fig. 1B). A wide range of IC_50_ values for MDM2 inhibitors in *TP53 WT* cell lines was previously reported^36^, similar to our observation with KRT-232 in B1011CL cells. All cell lines were then treated with both drugs for 72 h and with different dose ranges. Chou-Talalay methodology was used in order to obtain combination index (CI) values, where CI < 1 indicates synergistic activity, CI = 1 indicates additive activity, and CI > 1 indicates antagonistic activity. Selumetinib combined with KRT-232 demonstrated synergistic activity across all four cell lines tested and revealed a lower IC_50_ compared to single-agent treatment alone in all cell lines tested (Fig. 1C). Next, we assessed the colony forming capacity of these cell lines in presence of either single agent or the combination of selumetinib and KRT-232 by colony formation assay (CFA). We found that the combination of selumetinib and KRT-232 significantly affected anchorage-dependent growth and reduced colony formation capability in HCT116, LoVo, PDX.008CL, and B1011CL cells (Fig. 1D). Considering that long-term treatment might have a more profound effect on cells, we used CFA data and calculated IC_50_ of KRT-232 and selumetinib (Fig. 1E). All cell lines were sensitive to both drugs. Then we looked at the combination effect. The combination was synergistic at all dose ratios in PDX.008CL, at some dose ratios in B1011CL and HCT116, and was additive in LoVo (Fig. 1F).

### Selumetinib combined with KRT-232 affects in vitro cell cycle progression and demonstrates enhanced programmed cell death

**Figure 2:**
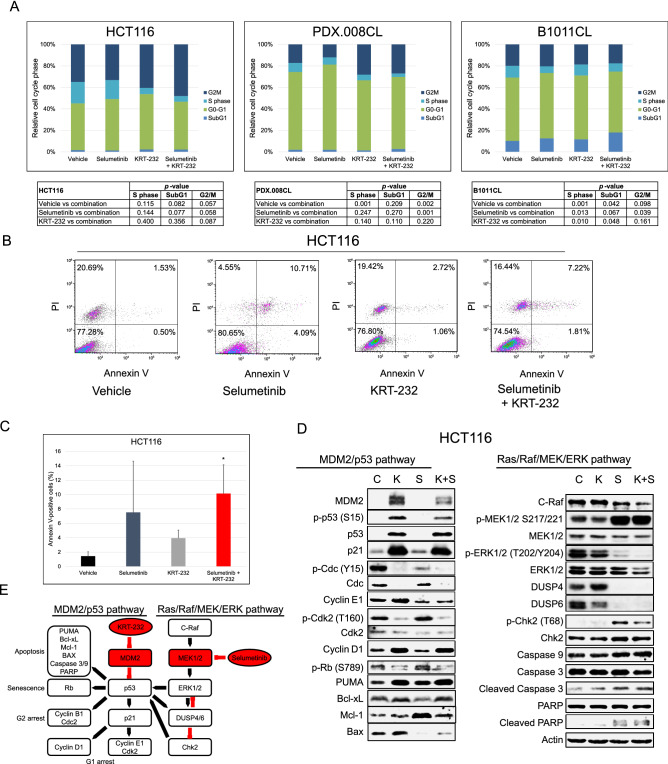
Effect of selumetinib combined with KRT-232 on cell cycle, apoptosis, and p53 and MAPK signaling. **(A)** Cell cycle analysis of two CRC cell lines (HCT116 and B1011CL) and one PTC cell line (PDX.008CL) after treatment for 24 h with vehicle, selumetinib (1 μM), KRT-232 (1 μM), or a combination of both. Percentages of cells in Sub-G1, G0-G1, S and G2/M phases of cell cycle are depicted. **(B)** Flow cytometry analysis of annexin-V positive cells in HCT116 colorectal cell line after treatment for 72 h by vehicle, selumetinib (1 µM), KRT-232 (1 µM) or combination of both. **(C)** Total percentage of annexin-V positive cell population shown in the graph after treatment for 72 h vehicle, selumetinib (1 µM), KRT-232 (1 µM) or combination of both. Values represent mean + /- SD. (*control vs combination, *p* = 0.046). **(D)** HCT116 CRC cells were treated with vehicle control (C), KRT-232 (1 μM, K), selumetinib (1 μM, S), or a combination of both (K + S) for 24 h. Cells were lysed and immunoblotting was performed using antibodies to identify changes in MDM2/p53 and MAPK pathways. Each blot had its loading control, in this panel one representative loading control (β-actin) was shown. The full-length blots are shown in Supplementary Fig. S2. **(E)** This illustration summaries the relation of antigens that were probed in **(D)**.

To determine the effects of the combination of KRT-232 and selumetinib on the cell cycle, we used HCT116, B1011CL, and PDX.008CL cells. We observed a significant reduction of cells in S phase in PDX.008CL and B1011CL with combination treatment compared to vehicle (*p* = 0.001) (Fig. 2A). With combination treatment, the percentage of B1011CL cells in S phase were increased compared to selumetinib (*p* = 0.013) and decreased compared to KRT-232 (*p* = 0.010) (Fig. 2A). We also found an increase in B1011CL sub-G1 population when cells were treated with the combination regimen compared to vehicle (*p* = 0.042) and KRT-232 (*p* = 0.048), respectively. PDX.008CL demonstrated a higher proportion of cells arrested in G2/M in the combination arm compared to vehicle (*p* = 0.002) and selumetinib (*p* = 0.001). B1011CL had a higher proportion of cells arrested in G2/M in the combination arm compared to selumetinib (*p* = 0.039).

KRT-232 was previously observed to induce apoptosis in sarcoma cell lines^20^. After demonstrating synergistic activity between selumetinib and KRT-232, we questioned whether apoptosis was enhanced with this combination. HCT116 cells were treated for 72 h with vehicle, selumetinib, KRT-232 or a combination of both agents. Cells were collected, stained with annexin V and propidium iodide, and analyzed using flow cytometry. A significantly higher rate of apoptosis was present in cells treated with selumetinib combined with KRT-232 compared to vehicle (1.4% vs 10.1%; *p* = 0.04) (Fig. 2B,C). However, we observed no statistically significant difference between both single agent and the combination regimen.

### Influence of selumetinib combined with KRT-232 on p53 pathway

We treated HCT116 cells with vehicle, KRT-232, selumetinib and their combination for 24 h. We lysed the cells and immunoblotted to observe the treatment effect on MDM2/p53 and Ras/Raf/MEK/ERK pathways (Fig. 2D). These signaling pathways interact with each other and both regulate apoptosis (Fig. 2E). MDM2/p53 pathway is involved in several cellular processes, such as cell cycle arrest at multiple points and senescence. Single agent KRT-232 treatment increased expression of MDM2 and p53. Likewise expression of p53 downstream proteins were regulated, such as an increase in p21 expression that regulates cell cycle, and an increase in Bax and PUMA expression that regulate apoptosis. We neither observed an increase in p-ERK expression nor MAPK activation as reported in other studies^31,32^. Selumetinib inhibited ERK1/2 phosphorylation, and DUSP4 and DUSP6 expression. There was an increase in expression of anti-apoptotic protein Mcl-1 and decrease in Mcl-1 downstream target, pro-apoptotic protein Bax, This was different from previous reports and where under similar conditions selumetinib had no effect on Mcl-1 expression^37,38^. Combination therapy had an additive effect and did not further enhance p53 activity. At this time point, we did not observe a significant increase in apoptosis either. Single agent selumetinib treatment resulted in a change in mobility of C-Raf and a significant increase in MEK1/2 phosphorylation. Inhibition of ERK1/2 phosphorylation released upstream proteins from negative feedback regulation^39^.

### Selumetinib combined with KRT-232 enhances in vivo antitumor efficacy in CRC PDX models compared with either single agent alone

**Figure 3:**
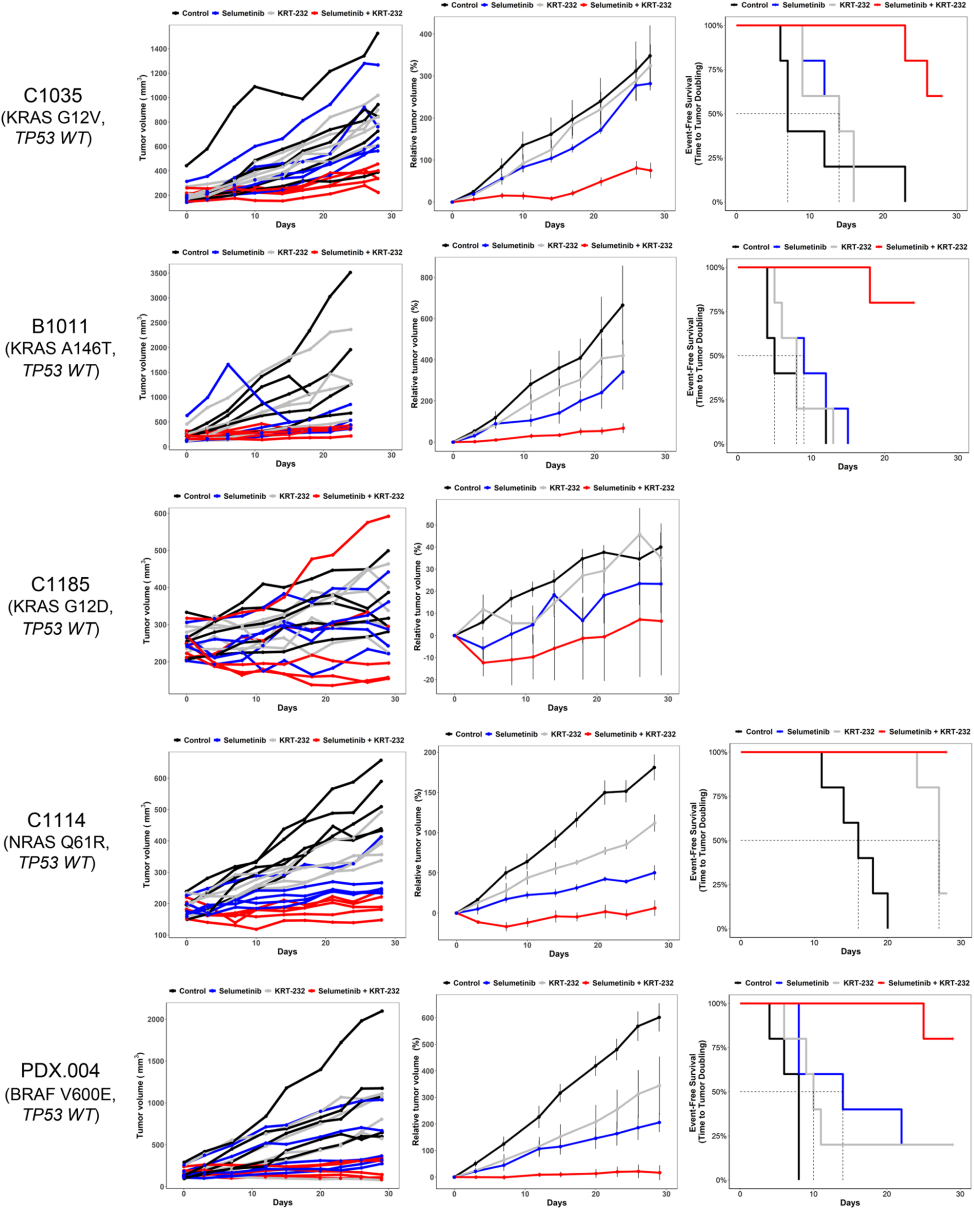
Selumetinib combined with KRT-232 inhibits tumor growth in CRC PDX models. Female athymic nu/nu mice bearing C1035 PDXs were treated with vehicle, selumetinib 15 mg/kg, KRT-232 15 mg/kg or the combination of both. The other four PDX were treated with vehicle, selumetinib 25 mg/kg, KRT-232 15 mg/kg or the combination of both. Selumetinib and KRT-232 were both administered daily. The treatment was stopped at 28 days or when tumor diameter reached 1.5 cm. Left panel, individual tumor volume change (mm^3^). Middle panel, growth curve graphs demonstrating mean change in tumor volume percentage ± SEM. Right panel, event-free survival curves. The C1185 model did not have any events and is not shown here.

To determine the in vivo antitumor efficacy of selumetinib combined with KRT-232, we evaluated five different CRC PDX models (C1035, B1011, C1185, C1114, and MDA-PDX.004 (PDX.004)) with different MAPK molecular characteristics (three *KRAS*, one *BRAF*, and one *NRAS* mutations). Figure 3 shows individual tumor volume (mm^3^) and relative tumor volume (%) change over time. Kaplan–Meier curves demonstrated event-free survival (EFS), assessing time to tumor doubling (Fig. 3). Four of five models (B1011, C1035, C1114, and PDX.004) showed EFS benefit of treatment with the combination compared to selumetinib alone (Table 1). In four out of five models (B1011, C1035, C1114, and PDX.004), tumor growth inhibition with combination treatment was greater than both single drugs (Table 2). C1185 model was different from the others and there was neither EFS benefit nor greater growth inhibition with combination treatment. Although the combination treatment inhibited tumor growth more than the control and other treatment groups in C1185 model, the variability in tumor sizes and particularly an outlier (the largest tumor was also treated by the combination) may have impacted the statistical analysis.

**Table 1:** Event-free survival **(**EFS) estimates of selumetinib-, KRT-232-, and combination-treated CRC and PTC PDX models.

Model	Treatment	N	No. events^a^	EFS median (days)^b^	EFS T/C^c^	Pairwise comparisons (p-value)^d^		
						Control	Selumetinib	KRT-232
B1011	Control	5	5	5				
	Selumetinib	5	5	9	1.80	0.222		
	KRT-232	5	5	8	1.60	0.348	0.510	
	Selumetinib + KRT-232	5	1	NA	>4.80	***0.002***	***0.002***	***0.002***
C1035	Control	5	5	7				
	Selumetinib	5	5	14	2.00	0.564		
	KRT-232	5	5	14	2.00	0.576	0.949	
	Selumetinib + KRT-232	5	2	NA	>4.00	***0.004***	***0.003***	***0.002***
C1114	Control	5	5	16				
	Selumetinib	5	0	NA	>1.75	***0.002***		
	KRT-232	5	4	27	1.69	***0.002***	***0.016***	
	Selumetinib + KRT-232	5	0	NA	>1.75	***0.002***	1.000	***0.016***
C1185	Control	5	0	NA				
	Selumetinib	5	0	NA	NA	1.000		
	KRT-232	5	0	NA	NA	1.000	1.000	
	Selumetinib + KRT-232	5	0	NA	NA	1.000	1.000	1.000
PDX.004	Control	5	5	8				
	Selumetinib	5	4	10	1.75	***0.029***		
	KRT-232	5	4	14	1.25	***0.032***	0.681	
	Selumetinib + KRT-232	5	1	NA	>3.63	***0.003***	***0.032***	***0.032***
PDX.008	Control	5	5	4				
	Selumetinib	5	5	10	2.50	***0.003***		
	KRT-232	5	5	5	1.25	***0.003***	***0.002***	
	Selumetinib + KRT-232	5	5	65	16.25	***0.003***	***0.005***	***0.002***
PDX.020	Control	5	5	15				
	Selumetinib	5	5	26	1.73	***0.002***		
	KRT-232	5	4	16	1.07	0.069	0.824	
	Selumetinib + KRT-232	5	1	NA	>2.80	***0.002***	***0.002***	***0.033***

^a^An event in each animal was defined as a doubling of tumor volume from initial tumor volume.

^b^EFS was defined as the time interval from initiation of study to the first event or to the end of the study period for tumors that did not double in volume.

^c^EFS T/C value was defined by the ratio of the median time to tumor doubling of the treatment group and the median time to tumor doubling of the control group. If the treatment group did not reach a doubling of tumor volume from initial tumor volume, then EFS T/C was defined as greater than the ratio of the last day of the study for the treatment group divided by the median time to tumor doubling for the control group.

^d^Log-rank test was used to compare survival distributions of two treatment groups. *p*-values < 0.05 were regarded as significant and are shown in bold italics. *NA* not available.

**Table 2:** Comparison of PDX tumor growth rates between groups.

Model	*p*-value						
	ANOVA	Selumetinib vs vehicle	KRT-232 vs vehicle	Combination vs vehicle	KRT-232 vs selumetinib	Combination vs selumetinib	Combination vs KRT-232
C1035	***<0.001***	0.650	0.903	***<0.001***	0.961	***0.001***	***<0.001***
B1011	***<0.001***	***0.027***	0.610	***<0.001***	0.298	***0.043***	***0.001***
C1185	0.052	0.688	0.988	0.071	0.861	0.464	0.134
C1114	***<0.001***	***<0.001***	***0.014***	***<0.001***	***0.026***	***<0.001***	***<0.001***
PDX.004	***0.002***	0.060	0.137	***<0.001***	0.976	***0.050***^***a***^	***0.020***
PDX.008	***<0.001***	***<0.001***	*0.003*	***<0.001***	***<0.001***	***<0.001***	***<0.001***
PDX.020	***<0.001***	***0.030***	*0.043*	***<0.001***	0.998	***0.015***	***0.010***

*p*-values < 0.05 were regarded as significant and are shown in bold italics.

^a^The *p*-value was 0.0495.

### Selumetinib combined with KRT-232 induces regression in PTC PDX models

**Figure 4:**
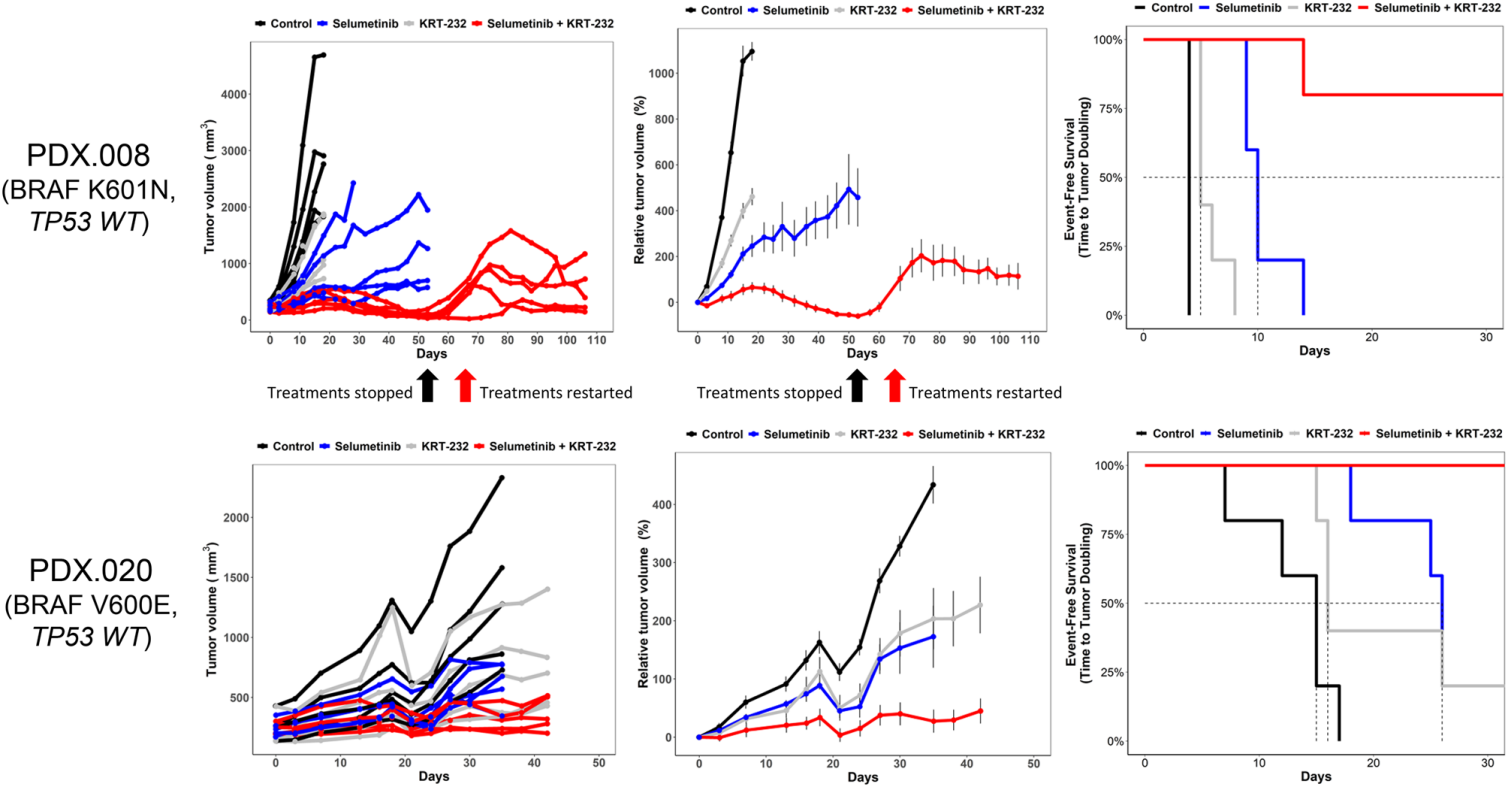
Selumetinib combined with KRT-232 inhibits tumor growth in PTC PDX models. Female athymic nu/nu mice bearing PTC PDXs were treated with vehicle, selumetinib 25 mg/kg, KRT-232 15 mg/kg, or the combination of both. Selumetinib and KRT-232 were both administered daily. Left panel, individual tumor volume change (mm^3^). Middle panel, growth curve graphs demonstrating mean change in tumor volume percentage ± SEM. Right panel, EFS curves. PDX.008: black arrow, complete regression on day 54, treatments stopped; red arrow, 100% increase in tumor size and treatments restarted on day 68.

To further investigate the in vivo effects of selumetinib plus KRT-232 across different histologies, we decided to utilize PTC PDX models. We tested two different PTC PDX models (MDA-PDX.008 (PDX.008) and PDX.020) both with presence of *BRAF* mutations (PDX.008 BRAF K601N and PDX.020 BRAF V600E). Significant antitumor efficacy was observed in both models. We observed complete regression of tumors in PDX.008 following 54 days of combination therapy. Dose interruption was introduced after 54 days of treatment, and mice were evaluated for regrowth of tumors. Treatments were reinitiated once tumors demonstrated 100% increase in tumor volume from baseline; this was observed 14 days after dose interruption. Despite tumor regrowth, tumors continued to respond to MDM2 combined with MEK inhibition upon rechallenge. Figure 4 shows individual tumor volume (mm^3^) and relative tumor volume (%) change in time and Kaplan–Meier curves demonstrating EFS. Both models showed EFS benefit with combination treatment compared with selumetinib or KRT-232 alone (Table 1). In both models, tumor growth inhibition with combination treatment was greater than both single drugs (Table 2). Neither significant weight loss nor other signs of toxicity were observed during the in vivo experiments.

### Combination of MDM2 and MEK inhibitors induces apoptosis and decreases cancer cell proliferation in vivo

**Figure 5:**
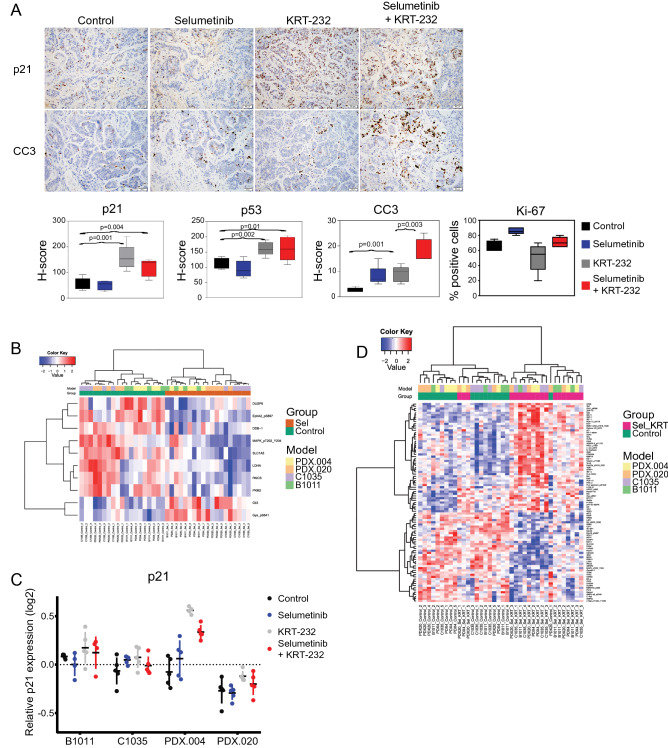
Selumetinib combined with KRT-232 induces apoptosis in vivo. **(A)** IHC analysis of PDX.004 CRC tumor samples obtained from the control, selumetinib, KRT-232, or combination treatment groups. (200 × magnification). Box plots of in vivo IHC H-score of p21, p53, and cleaved caspase 3 (CC3), and percentage of Ki-67–positive tumor cells. Values represent mean ± SD. Statistical significance was determined using the Student’s t-test. **(B)** Heatmap generated (with false discovery rate [FDR] 0.05) from RPPA samples obtained from selumetinib-only treated PDXs vs untreated controls demonstrated 10 differentially expressed proteins (DEPs), where samples were clustered by the treatment. **(C)** Scatter plot created from RPPA samples obtained from the control, selumetinib, KRT-232, or combination treatment groups demonstrating the relative expression of p21. Bars show mean ± SD. **(D)** Heatmap generated (with FDR 0.05) from RPPA samples obtained from PDXs treated with selumetinib plus KRT-232 vs untreated controls demonstrated 91 DEPs; samples were clustered by treatment.

Evidence of enhanced induction of apoptosis was observed with the combination of the MDM2 inhibitor with MEK inhibitor in our PDX.004 model using immunohistochemical (IHC) analysis for cleaved caspase 3 (average percentage of positive tumor cells: control, 2.6%; selumetinib, 8.2%; KRT-232, 9%; and combination, 18% (combination vs selumetinib, *p* = 0.003, combination vs KRT-232, *p* = 0.003; Fig. 5A). Compared with the control, combination treatment increased cleaved caspase 3 expression in *BRAF* mutant PDX.004 and PDX.020 models (Supplementary Fig. S1).

Tumor samples were additionally evaluated for p53 and p21 expression by analyzing H-scores obtained from IHC. Similar to what we observed in vitro, we found both p53 and p21 to be upregulated, mainly in the KRT-232 single-agent (KRT-232 vs control, p21 *p* = 0.001 and p53 *p* = 0.002) and combination (combination vs control, p21 *p* = 0.004 and p53 *p* = 0.01) treatment groups (Fig. 5A). We also looked at Ki-67–positive cells as a measure of proliferation and found no difference among groups. Compared with the control, combination treatment decreased Ki-67 expression in *KRAS* mutant B1011 and C1035 models, however, even in these two models the percent of Ki-67-positive cells remained high (Supplementary Fig. S1).

To assess the impact of therapy on the functional proteomic profile, we performed RPPA on PDXs treated with selumetinib alone, KRT-232 alone, and the selumetinib/KRT-232 combination and untreated controls in four different models: CRC models B1011, C1035, and PDX.004 and PTC model PDX.020. Differentially expressed proteins (DEPs) are shown in Supplementary Table S1. Comparing selumetinib-treated PDXs with controls, we identified 10 DEPs, including downstream targets of MEK1/2: p-ERK1/2 Thr202/Tyr204 and dual-specificity phosphatase 6 (DUSP6)^40,41^. We corrected the random effect of models and generated a heatmap for the DEPs, in which the samples were clustered by treatment (Fig. 5B). When we compared KRT-232–treated PDXs with controls in all four models together, the only DEP was p21 (*q* = 0.015). The increase in p21 on RPPA was consistent with our finding of p21 upregulation in IHC data (Fig. 5A). When individual models were considered, difference in p21 expression was significant only in PDX.004 (*q* = 0.0013). Relative p21 expression in all models and treatment groups is shown in Fig. 5C. In PDX.004, p21 was significantly increased with KRT-232 (*p* < 0.001) and combination treatment (p < 0.001) but not with selumetinib treatment (*p* = 0.369). The difference between KRT-232 and combination treatment groups was not significant (*p* = 0.089). When we compared the PDXs treated with the combination of KRT-232 and selumetinib to controls, we identified 91 DEPs (Fig. 5D). Ingenuity Pathway Analysis identified p53 as the top upstream regulator (*p* = 1.96e−26).

## Discussion

Although MAPK pathway alterations are among the most common genomic alterations, genomically informed therapeutic options are largely lacking. In this study, after inhibition of the MAPK pathway by selumetinib (MEK1/2 inhibitor) and stabilization of p53 pathway by KRT-232 (MDM2 inhibitor), we observed significantly increased growth inhibitory activity both in vitro and in vivo in CRC and PTC with MAPK alterations and *TP53 WT*. This confirmed our hypothesis that dual MEK and MDM2 inhibition will yield significant tumor growth inhibition in genomically matched colorectal and thyroid cancers. In PDX models of colorectal and thyroid cancers, we observed significant inhibitory effect of the combination of selumetinib and KRT-232 on tumor growth across a spectrum of MAPK genomic backgrounds, including *BRAF*, *KRAS*, and *NRAS* mutations, compared to single-agent treatment with either drug.

BRAF or MEK inhibition alone in *KRAS-* and *BRAF*-mutant CRCs have shown limited activity^42^, likely because of MAPK pathway reactivation through adaptive responses^43^. However, Kopetz et al. showed that the combination of MEK with BRAF and EGFR inhibitors improved overall survival compared with doublet or single-agent treatment, highlighting the importance of developing combination therapies^44^. Our *BRAF*-mutated models demonstrated the highest level of sensitivity to combined therapy of selumetinib and KRT-232 and greatest tumor reduction, especially in our PDX.008 model (which harbors an activating BRAF K601N mutation), despite introduction of a hiatus and re-challenging with treatment. *BRAF* mutations in codon 601 are located in the kinase domain and have been shown to induce cell proliferation^45^. Recently it has been shown that KRT-232 inhibits tumor growth in *BRAF WT* melanoma PDXs, and KRT-232 in combination with BRAF or MEK inhibitors showed a synergistic effect in BRAF V600–mutant tumors^46^. A similar synergistic effect was observed with MEK inhibitor trametinib and three MDM2 inhibitors (nutlin-3, RG7388, and HDM201) in BRAF V600E-mutant and *TP53 WT* melanoma^47^. Thus, further study is needed to determine whether MEK/MDM2 inhibition can be an option for tumors that have progressed on BRAF inhibitors and whether a BRAF/MEK/MDM2 inhibitor triplet would be an option to deepen and prolong responses in BRAF V600–mutant tumors.

MDM family members, MDM2 and MDM4, partner in their action, and both negatively regulate p53. In our study, we limited our experiments to models with *TP53 WT*. We thus cannot comment on expected results with this combination with *TP53* mutant models. Stabilization of *TP53 WT* resolves DNA damage or induces apoptosis to protect cells from becoming tumorigenic. However, we cannot exclude the possibility that stabilization of mutant *TP53* may have the opposite effect, promoting growth, metastatic properties, and genetic instability^48^. *TP53 WT* status is essential for KRT-232 to robustly exert its antitumor effects by blocking the interaction of p53 and MDM2. In nearly 8% of CRCs with *TP53 WT*, tumorigenesis is driven by MDM2 amplification and/or overexpression^49^. Being mutually exclusive to *TP53* mutations, MDM2 gene amplification has been shown to be associated with chromosome 12 gain^49^, resulting in both RNA and protein overexpression^50,51^, followed by downregulation of p53 and induction of chemoresistance^14^. Amplification is a major cause of MDM2 overexpression; however, other mechanisms have been shown to induce MDM2 overexpression, including SNP309 germline mutation^52^ and MDM2 promoter binding by estrogen in hormone-positive breast cancers^53^. However, these tumors did overexpress MDM2 to the level of MDM2-amplified tumors^19^. Our findings, including immunoblotting of the p53 pathway, demonstrated a strong p53 reactivation in both single-agent KRT-232 and combination treatment, which induced apoptosis. Cell cycle analysis demonstrated variability in response, with decreases in S-phase and increases in G2/M cell populations in both KRT-232 and combination treatment groups in two of three cell lines, and there was no statistical significance between them. These findings, combined with upregulation of p53 and p21, suggest the effect on cell cycle is mainly driven by p21 through reactivation of p53 by KRT-232 and/or combination therapy. The increase in G2/M cell populations observed is most likely driven by KRT-232 in both single-agent and combination treatment arms. A similar finding was observed with MDM2 inhibitors and was found to be due to double-stranded DNA breaks’ inducing a DNA damage response and blocking the entry of cells into mitosis^54^. Longer treatment and combining the compounds based on their potencies could reveal significant results in cell cycle analysis. In addition to the combination, both single agents induced apoptosis in vitro. KRT-232 increases p53 and p21 expression, which is followed by cell cycle arrest in G1 until apoptosis is induced^55^. MEK inhibitors, including selumetinib, inhibit ERK, and ERK inhibition induces the p53 upregulated modulator of apoptosis (PUMA)^56^, resulting in p53-independent apoptosis. In vivo data supported this observation. The expression of p53 and p21 were increased in the KRT-232 and combination treatment groups, yet cleaved caspase 3 was increased in all treatment groups, including the selumetinib group.

The RPPA result also produced interesting observations. The DEPs in the combination treatment vs control groups were involved in different processes, such as induction of apoptosis (Bim, Bax, IRF-1/3 upregulation, PARP1 downregulation), cell cycle arrest (p21 and p-Chk1 upregulation), cellular stress (XBP1 upregulation), fatty acid synthesis (FAS downregulation), T-cell signaling (Zap70 and Vav1 upregulation), and resistance to BRAF inhibitors (EPHA2 downregulation). Several mechanisms could be involved in drug response, which warrant further study to understand their significance. There may also be differences in the downstream effects of MEK and MDM2 inhibition including differential effects on proliferation and apoptosis. Further work is needed to determine whether downstream effects differ by genotype, histology and co-alterations and to gain deeper mechanistic insights into combination therapy efficacy observed.

Major mechanisms of resistance to MDM2 inhibitors include *TP53* mutations or activation of IGFBP1-ERK signaling^31,57^. MEK inhibition resistance is mediated through activation of the PI3K pathway or FGFR signaling^58,59^. Addition of a MEK inhibitor to a MDM2 inhibitor overcame MAPK-mediated resistance to MDM2 inhibition^31^. Acquired resistance to combined MDM2 and MEK inhibition has been previously observed in colon and non-small cell lung cancer cells^60^. In our study, we did not do analysis of acquired resistance.

Limitations of our study include absence of genomic analysis of treated PDXs to determine if acquired *TP53* mutations were detected; however, the tumors remained sensitive to re-challenge with combination therapy after re-growth in PDX.008, suggesting that the residual cancer cells were still *TP53 WT*. As these combinations advance to the clinic, further studies will be needed to identify acquired resistance mechanisms.

Our preclinical findings demonstrate synergistic and enhanced antitumor efficacy of the combination of MDM2 and MEK inhibition in MAPK-altered, *TP53 WT* tumors of colorectal and thyroid origin. These results support further investigation of the MDM2/MEK combination in tumors of epithelial origin in the clinic.

## Methods

### Cell lines and cultures

HCT116 and LoVo CRC cell lines were acquired from American Tissue Culture Collection (Manassas, VA, USA). MDA-PDX.008CL PTC and B1011CL CRC cell lines were generated from MDA-PDX.008 PTC and B1011 CRC PDXs, respectively. Samples were collected with informed consent under an Institutional Review Board–approved protocol for PDX generation, as described below. PDXs were collected in DMEM/F12 supplemented with 10% fetal bovine serum, chopped into 1–2 mm pieces, and incubated overnight on a shaker at 37 °C and 200 rpm. Cells were filtered through a 100 µm nylon mesh and plated in T25 ultra-low attachment flasks. Three months later, cells were further transferred into cell culture plates. Fidelity confirmation of PDX-derived cell lines was completed using short tandem repeat analysis. HCT116 cells were cultured in RPMI-1640 supplemented with 10% fetal bovine serum, and LoVo cells were cultured in F12K medium (Kaighn’s Modification of Ham’s F-12 Medium) with 10% fetal bovine serum at 37 °C and humidified in 5% CO_2_.

### Reagents

KRT-232 (Kartos Therapeutics, Redwood City, CA, USA) and selumetinib (AstraZeneca, Cambridge, UK) were obtained through National Cancer Institute Cancer Therapy Evaluation Program. KRT-232 was prepared in 0.5% methylcellulose, 1% Tween 80 and water, while selumetinib was prepared in 0.5% methylcellulose and water for in vivo studies. Dimethyl sulfoxide was obtained from Sigma-Aldrich (St. Louis, MO, USA). All drugs used for in vitro studies were prepared in dimethyl sulfoxide.

### Cell growth assays

SRB assay was used to determine the anti-proliferative activity and IC_50_^61^. Cells were seeded in 96-well plates at a density of 5000 cells per well based on cell line growth characteristics. The IC_50_ values were determined based on a dose–response curve generated using CalcuSyn Software, v2.11 (Biosoft, Cambridge, UK). The drugs were combined with a fixed ratio in all cell lines, whereas the selumetinib-to-KRT-232 ratios in each model were: PDX.008CL, 5:1; HCT116, 6:1; B1011Cl, 0.8:1; and LoVo, 0.5:1. Chou-Talalay methodology was used in order to obtain Combination Index (CI) values and, where CI < 1 indicates synergistic activity, CI = 1 indicates additive activity and CI values > 1 indicates antagonistic activity. Experiments were performed at least three times.

### Colony formation assay

Live cells were counted using trypan blue and were seeded at a density of 1 × 10^3^ for HCT116, LoVo, and PDX.008CL and 5 × 10^3^ for B1011CL in each well on 60-mm plates in duplicate for each treatment group. Cells were treated the following day. Culture medium and drugs were changed twice a week. Cells were cultured for 10 days to three weeks. The colonies were fixed in 10% formalin and stained with 0.05% crystal violet in 25% methanol. Total colony area were measured using ImageJ v1.52a software (NIH, Bethesda, MD, USA). Individual IC_50_ values were calculated using CalcuSyn. The Bliss independence model was used to evaluate the drug interactions across all dose combinations tested^62^. Interaction index values < 1, indicate synergistic activity, values = 1 indicate additive activity, and values > 1 indicate antagonistic activity.

### Western blot assay

Immunoblotting was performed with the following antibodies: Bax (D2E11, #5023), Bcl-xL (#2762), Caspase-3 (#9662), Caspase-9 (#9502), p-Cdc2 (Tyr15) (#9111), Cdc2 (POH1, #9116), p-CDK2 (Thr160) (#2561), p-Chk2 (Thr68) (C13C1, #2197), Chk2 (#2662), c-Raf (#9422), CDK2 (78B2, #2546), Cyclin D1 (#2922), Cyclin E1 (D7T3U, #20808), DUSP4 (D9A5, #5149), DUSP6 (#39441), p-Erk1/2 (Thr202/Tyr204) (D13.14.4E, #4370), Erk1/2 (137F5, #4695), Mcl-1 (D5V5L, #39224), MDM2 (D1V2Z, #86934), p-MEK1/2 (Ser217/221) (41G9, #9154), MEK1/2 (47E6, #9126), p21 (12D1, #2947), p-p53 (Ser15) (16G8, #9286), p53 (7F5, #2527), PARP (46D11, #9532), PUMA (D30C10, #12450), p-Rb (Ser789) (#9307), (all from Cell Signaling Technology (Boston, MA, USA)), Actin (C-2, #sc-8432 AF790, Santa Cruz Biotechnology), and β-actin antibody (AC-15, #A5441, Sigma-Aldrich). Immunoblotting signals were visualized using an Odyssey IR imaging system (Li-Cor Biosciences, Lincoln, NE, USA). Image Studio software v4.0 was used for analysis of the bands.

### Annexin V assay

Cell were treated for 72 h. Both floating cells and attached cells were collected and probed for apoptosis using an Annexin V/PI Apoptosis kit (Roche, Indianapolis, IN, USA) according to the manufacturer’s protocol. Labeled cells were analyzed by flow cytometry in The University of Texas MD Anderson Cancer Center (MDACC) Flow Cytometry and Cellular Imaging Core Facility.

### Cell cycle analysis

Cells were plated and treated the following day in duplicates for 24 h with either vehicle, selumetinib 1 µM, KRT-232 1 µM, or both. Samples were collected, fixed, and analyzed by flow cytometry in the MDACC Flow Cytometry and Cellular Imaging Core Facility using propidium iodide staining kit (Abcam, Cambridge, MA, USA). We evaluated the percentages of cells in each cell cycle phase: sub-G1, G0-G1, S, and G2/M.

### In vivo studies

For PDX generation, the human tumor sample collection was approved by the MD Anderson Cancer Center (MDACC) Institutional Review Board (IRB protocol #PA13-0592 and LAB10-0982). All methods involving human studies were carried out in accordance with the ethics principles of the Declaration of Helsinki and the International Council of Harmonization Guidelines on Good Clinical Practice. A written informed consent was obtained from all participating patients. All animal experiments were approved by MDACC’s Animal Care and Use Committee (protocol #00001405-RN01), which is accredited by the Association for Assessment and Accreditation of Laboratory Animal Care (AAALAC). All animal experiments were performed in accordance with the approved protocol, in accordance with the Institutional Animal Care and Use Committee (IACUC) guidelines. Animal experiments were done, analyzed, and presented in accordance with Animal research: Reporting in vivo experiments (ARRIVE) guidelines.

Subcutaneous tumors were implanted into 6- to 8-week-old female athymic BALB/c nu/nu mice. Treatments began when tumor diameter reached between 150 and 200 mm^3^. The mice were euthanized at the end point of the study (at 28 days or when tumor diameter reached 1.5 cm). Tumor volume was obtained by using: tumor volume (mm^3^) = (width × width × length)/2. Tumor volumes and weights were measured twice a week.

In order to determine in vivo antitumor efficacy of selumetinib in combination with KRT-232, mice were randomized into four groups. C1035 xenografts were treated with vehicle, KRT-232 15 mg/kg, selumetinib 15 mg/kg, and combination of both (n = 5). The other six xenografts models were treated with vehicle, KRT-232 15 mg/kg, selumetinib 25 mg/kg, and combination of both (n = 5). Both selumetinib and KRT-232 were dosed daily and administered through oral route.

### Sample evaluation

Tumors were harvested 4 h after last treatment. Half of the tumor was placed in formalin and the other half was snap frozen. Hematoxylin and eosin stains from formalin-fixed and paraffin-embedded PDX samples were reviewed by a board-certified pathologist for the presence of tumor cells. All but one sample had sufficient (> 100 tumor cells) tumor for immunohistochemistry (IHC) evaluation. The percentage of necrosis within the tumor was estimated on the hematoxylin and eosin stain.

### Reverse-phase protein array

Reverse-phase protein array (RPPA) was performed at MDACC Functional Proteomics core facility as previously described^63^. DEPs and their association with canonical pathways were analyzed using Ingenuity Pathway Analysis software (Qiagen, Hilden, Germany).

### Immunohistochemistry

From each sample, 4-µm sections were stained with antibodies against Ki-67 (clone MIB-1, code M7240, Dako/Agilent, Glostrup, Denmark), cleaved PARP (Asp214; clone: D64E10, #5625), cleaved caspase 3 (Asp175; #9661, Cell Signaling Technology), p21 (clone: F5, #sc-6246, Santa Cruz, Dallas, USA), and p53 (clone: DO-7, Leica Biosystems, Wetzlar, Germany). For Ki-67, cleaved PARP, and cleaved caspase 3, the percentage of positive tumor cells was estimated regardless of staining intensity. For p21 and p53, the percentage of positive cells as well as the intensity of staining was evaluated, resulting in an H-score (range: 0–300). Necrotic areas were excluded from evaluation.

### Bioinformatics analysis

The RPPA data were normalized for protein loading and transformed to log2 values for quality control and comparison analysis. The comparisons were done by fitting the linear mixed effect models using treatment/control as the fixed effect and model as the random effect. To control the false discovery rate, we calculated the q-value for each protein in each comparison and used the q-values to identify the DEPs.

### Statistics

For in vitro studies, two-group comparisons were done using the Student’s t-test and p < 0.05 was considered statistically significant. The normalized log-transformed RPPA expression values were used for the analysis. Boxplot, unsupervised hierarchical clustering and Principal Component Analysis were used for the quality assessment. The comparisons were done by fitting linear mixed effect model (LMEMs) using "Group" as the fixed effect and "Model" as the random effect. A beta-uniform mixture (BUM) model was used to fit the p-values from LMEMs and identified the differentially expressed proteins (DEPs) with specified FDR.

For in vivo studies, the % tumor volume change per time point was calculated as a relative level of tumor growth change from baseline: ${\delta }_{t}=\frac{{V}_{t}-{V}_{0}}{{V}_{0}}\times 100$, where ${V}_{t}$ is the tumor volume a time $t$ and ${V}_{0}$ is the tumor volume at baseline. An event in each animal was defined as a doubling of tumor volume from initial tumor volume. EFS was defined as the time interval from initiation of study to the first event or to the end of the study period for tumors that did not doubling in volume. The time to event was determined using linear interpolation based on the following formula: for animals for which there was no tumor volume measurement at time t but which had flanking volume measurements at time ${t}_{0}$ and ${t}_{1}$ such that ${t}_{0}<t<{t}_{1}$, we used linear interpolation to compute the measurement. That is, we computed ${\Delta }_{{V}_{t}}={\Delta }_{{V}_{0}}+\beta \left(t-{t}_{0}\right)$, where $\beta =({\Delta }_{{V}_{t1}}-{\Delta }_{{V}_{t0}})/({t}_{1}-{t}_{0})$. Using the Kaplan–Meier survival estimates, an EFS T/C value was defined by the ratio of the median time to tumor doubling of the treatment group and the median time to tumor doubling of the control group. If the treatment group did not reach a doubling of tumor volume from initial tumor volume, then EFS T/C was defined as greater than the ratio on the last day of the study for the treatment group divided by the median time to tumor doubling for the control group. Tumor growth curves were analyzed using a linear mixed model. A mixed effects model was fit, with treatment, time and their interaction as fixed-effects parameters, and the time trend for each subject as random effects^64^. The log-rank test was used to compare survival distribution of two treatment groups. All statistical analyses were performed using R software version 3.5.2. A *p*-value < 0.05 was regarded as significant.

## Supplementary Information

Supplementary Information.
