# Supplementary material for: Combined MEK/MDM2 inhibition demonstrates antitumor efficacy in TP53 wild-type thyroid and colorectal cancers with MAPK alterations
Source: Sci Rep. 2022 Jan 24;12:1248. doi: 10.1038/s41598-022-05193-z (PMC8786858; doi:10.1038/s41598-022-05193-z)
Supplement: Supplementary file 1 — Supplementary Information. [file 41598_2022_5193_MOESM1_ESM.pdf]

Combined MEK/MDM2 Inhibition Demonstrates Antitumor Efficacy in *TP53* Wild-Type Thyroid and Colorectal Cancers with MAPK Alterations

Seyed Pairawan, Argun Akcakanat, Scott Kopetz, Coya Tapia, Xiaofeng Zheng, Huiqin Chen, Min Jin Ha, Yasmeen Rizvi, Vijaykumar Holla, Jing Wang, Kurt W. Evans, Ming Zhao, Naifa Busaidy, Bingliang Fang, Jack A. Roth, Ecaterina Ileana Dumbrava, Funda Meric-Bernstam.

Supplementary Information

Supplementary Table S1. Differentially expressed proteins. RPPA was used to identify differentially expressed proteins in xenograft tumors between vehicle control and the three treatment groups (selumetinib, KRT-232, and their combination) in four different PDX models: CRC models B1011, C1035, PDX.004, and PTC model PDX.020. Relative expression values and adjusted *p*-values are shown.

Supplementary Figure S1. Evaluation of Ki-67 and cleaved caspase 3 (CC3) expression in PDX tumors by immunohistochemistry using percentage and H-score, respectively. Each box extends from 25th to 75th percentiles, and the line in the middle is plotted at the median. The whiskers show minimum and maximum values. The Student's t-test was used to compare control and combination treatment groups.

Supplementary Figure S2. Full-blots. A molecular weight marker was loaded on each gel. Following immunoblotting, blots were cut to three parts and each part was probed with a different antibody. On some of the cut blots, we were not able to show the molecular weight marker.

Supplementary Table S1. Differentially expressed proteins in all four models

Control vs selumetinib			
	Control.mean	Sel.mean	p.adj
SLC1A5	0.13	-0.21	0.0008
EphA2_pS897	0.29	-0.04	0.0008
Gys_pS641	-0.08	0.24	0.0189
Gli3	-0.09	0.12	0.0189
MAPK_pT202_Y204	0.28	-0.02	0.0191
PAICS	0.14	-0.09	0.0268
LDHA	0.22	-0.13	0.0316
PKM2	0.13	-0.06	0.0438
DDB-1	0.05	-0.10	0.0449
DUSP6	0.09	-0.04	0.0497

Control vs KRT-232			
	Control.mean	KRT232.mean	p.adj
p21	-0.09	0.15	0.0150

Control vs selumetinib + KRT-232			
	Control.mean	Sel_KRT.mean	p.adj
SLC1A5	0.13	-0.32	0.0001
PAICS	0.14	-0.15	0.0005
EphA2_pS897	0.29	-0.06	0.0005
EphA2	0.21	-0.15	0.0007
MAPK_pT202_Y204	0.28	-0.05	0.0008
IRF-3	-0.08	0.05	0.0011
CDT1	0.09	-0.08	0.0011
IRF-1	-0.11	0.19	0.0013
XBP-1	-0.48	0.54	0.0017
Erk5	-0.04	0.19	0.0024
Slfn11	-0.11	0.08	0.0026
Tuberin_pT1462	-0.05	0.07	0.0032
FN14	0.06	-0.08	0.0032
Gys_pS641	-0.08	0.23	0.0057
VEGFR-2_pY1175	-0.04	0.12	0.0057
LDHA	0.22	-0.19	0.0060
p44-42-MAPK	-0.08	0.10	0.0061
TAZ	-0.05	0.21	0.0061
ZAP-70	-0.08	0.55	0.0061
BMK1-Erk5_pT218_Y22	-0.04	0.08	0.0075
UVRAG	0.04	-0.12	0.0075
HSP60	0.13	-0.15	0.0075
RRM2	0.11	-0.06	0.0075
PLC-gamma1_pS1248	-0.06	0.07	0.0076
PARP	0.12	-0.32	0.0100
Gli3	-0.09	0.21	0.0100

FoxO3a_pS318_S321	-0.09	0.06	0.0100
DUSP6	0.09	-0.04	0.0100
Tyro3	-0.07	0.05	0.0116
CDKN2A	-0.04	0.12	0.0116
Calnexin	0.16	-0.08	0.0116
Cdc6	-0.03	0.09	0.0121
ZEB1	-0.05	0.08	0.0122
TUFM	-0.06	0.04	0.0122
TFRC	0.21	-0.26	0.0122
SFRP1	-0.04	0.08	0.0124
DUSP4	0.41	-0.03	0.0142
PREX1	0.05	-0.07	0.0145
AMPK-a2_pS345	0.04	-0.07	0.0145
RRM1	0.01	-0.08	0.0145
4E-BP1	0.12	-0.01	0.0151
MERIT40_pS29	0.06	-0.08	0.0154
MMP14	-0.13	0.05	0.0157
YES1	-0.05	0.04	0.0160
Chk1_pS296	-0.04	0.31	0.0162
AMPKa	0.07	-0.08	0.0162
PKA-a	0.03	0.30	0.0162
c-Jun_pS73	-0.05	0.06	0.0174
MITF	-0.06	0.04	0.0180
PEA-15	-0.07	0.10	0.0184
Histone-H3_pS10	-0.02	0.08	0.0185
BRD4	0.26	-0.19	0.0185
p21	-0.09	0.06	0.0186
CD38	-0.07	0.06	0.0186
Bax	-0.01	0.13	0.0189
IGFBP2	1.01	0.55	0.0197
b-Actin	-0.04	0.15	0.0200
MYH11	-0.19	0.32	0.0210
VAV1	-0.16	0.31	0.0219
Akt	-0.10	0.11	0.0220
PEA-15_pS116	-0.08	0.09	0.0220
S100A4	0.02	-0.08	0.0233
MCT4	0.10	-0.20	0.0233
DDR1	-0.04	0.06	0.0233
MelanA	0.02	-0.06	0.0238
N-Cadherin	-0.02	0.09	0.0238
HSP27_pS82	0.11	0.47	0.0239
Bim	0.06	0.33	0.0247
Notch1	0.09	-0.05	0.0247
CIITA	0.03	-0.04	0.0247
c-Met_pY1234_Y1235	0.01	-0.09	0.0247
TFAM	0.13	-0.18	0.0250
LAD1	0.03	-0.18	0.0256
IRS2	0.03	-0.20	0.0291
Ets-1	-0.06	0.04	0.0309

PRC1_pT481	-0.05	0.04	0.0310
HLA-DQA1	-0.03	0.05	0.0316
FASN	0.30	-0.09	0.0330
SGK3	-0.03	0.11	0.0359
ER-a	0.03	-0.06	0.0359
WIPI1	0.16	-0.12	0.0359
eEF2	0.11	-0.04	0.0408
Claudin-7	0.08	-0.20	0.0408
S6_pS235_S236	0.22	-0.28	0.0408
VHL	-0.02	0.07	0.0408
LC3A-B	0.00	0.11	0.0444
C-Raf	0.07	0.00	0.0465
Myosin-IIa	-0.08	0.12	0.0482
GCLC	0.16	-0.03	0.0494
Hif-1-alpha	-0.01	0.06	0.0494
Stat5a	-0.08	0.08	0.0497

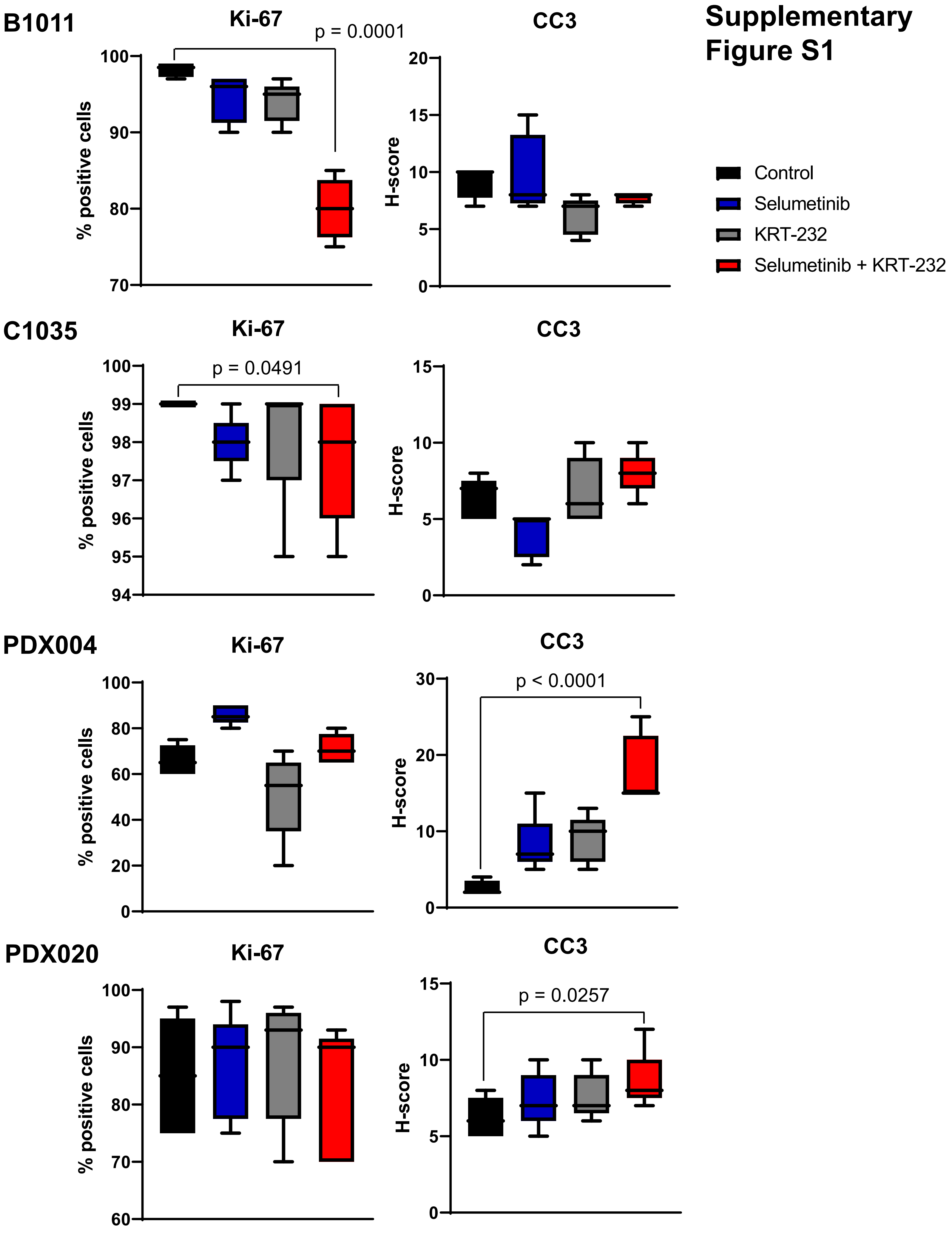

Supplementary Figure S1
B1011
Ki-67
p = 0.0001
% positive cells
100
90
80
70
CC3
H-score
20
15
10
5
0
Control
Selumetinib
KRT-232
Selumetinib + KRT-232
C1035
Ki-67
p = 0.0491
% positive cells
100
99
98
97
96
95
94
CC3
H-score
15
10
5
0
PDX004
Ki-67
% positive cells
100
80
60
40
20
0
CC3
p < 0.0001
H-score
30
20
10
0
PDX020
Ki-67
% positive cells
100
90
80
70
60
CC3
p = 0.0257
H-score
15
10
5
0

Supplementary Figure S2

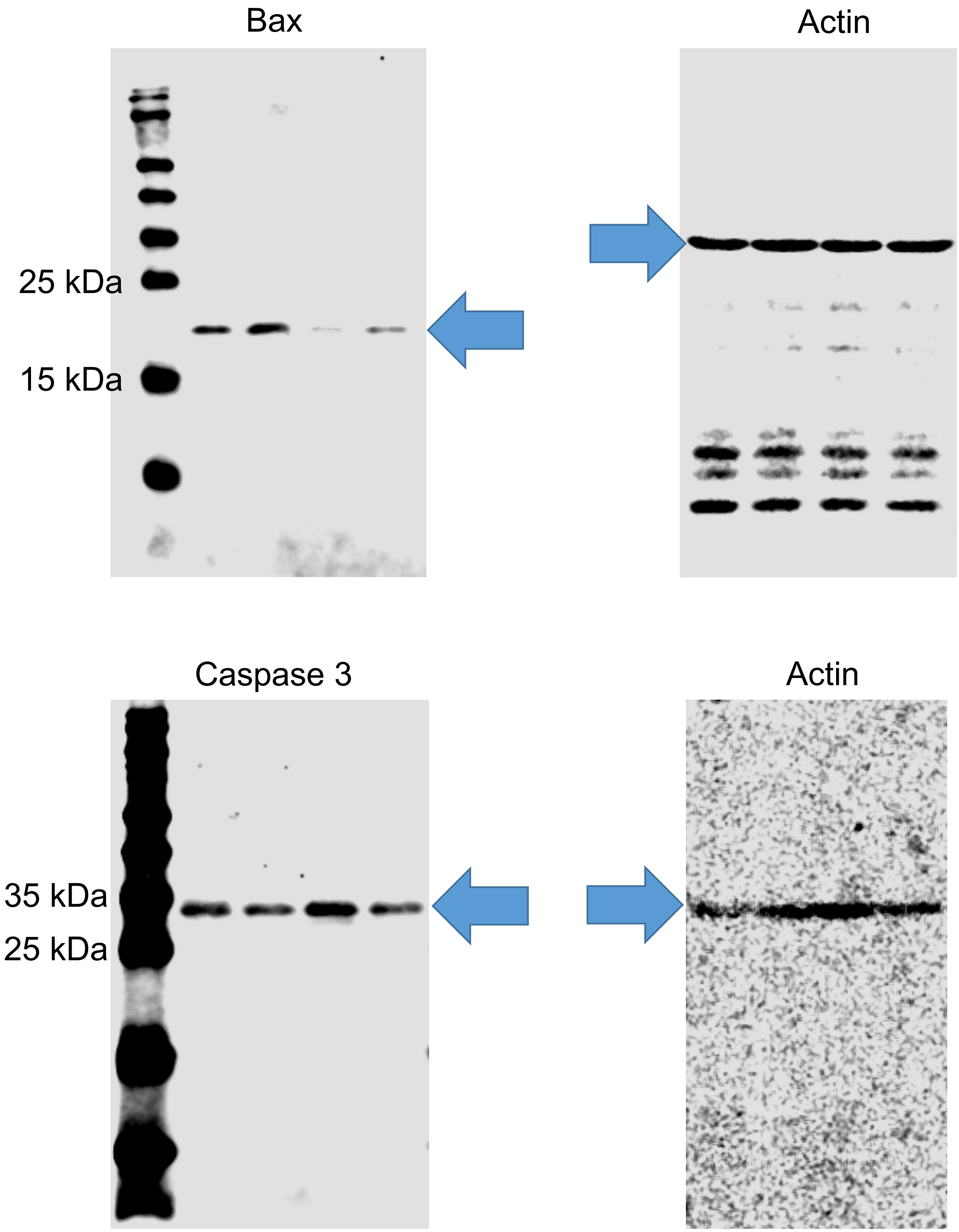

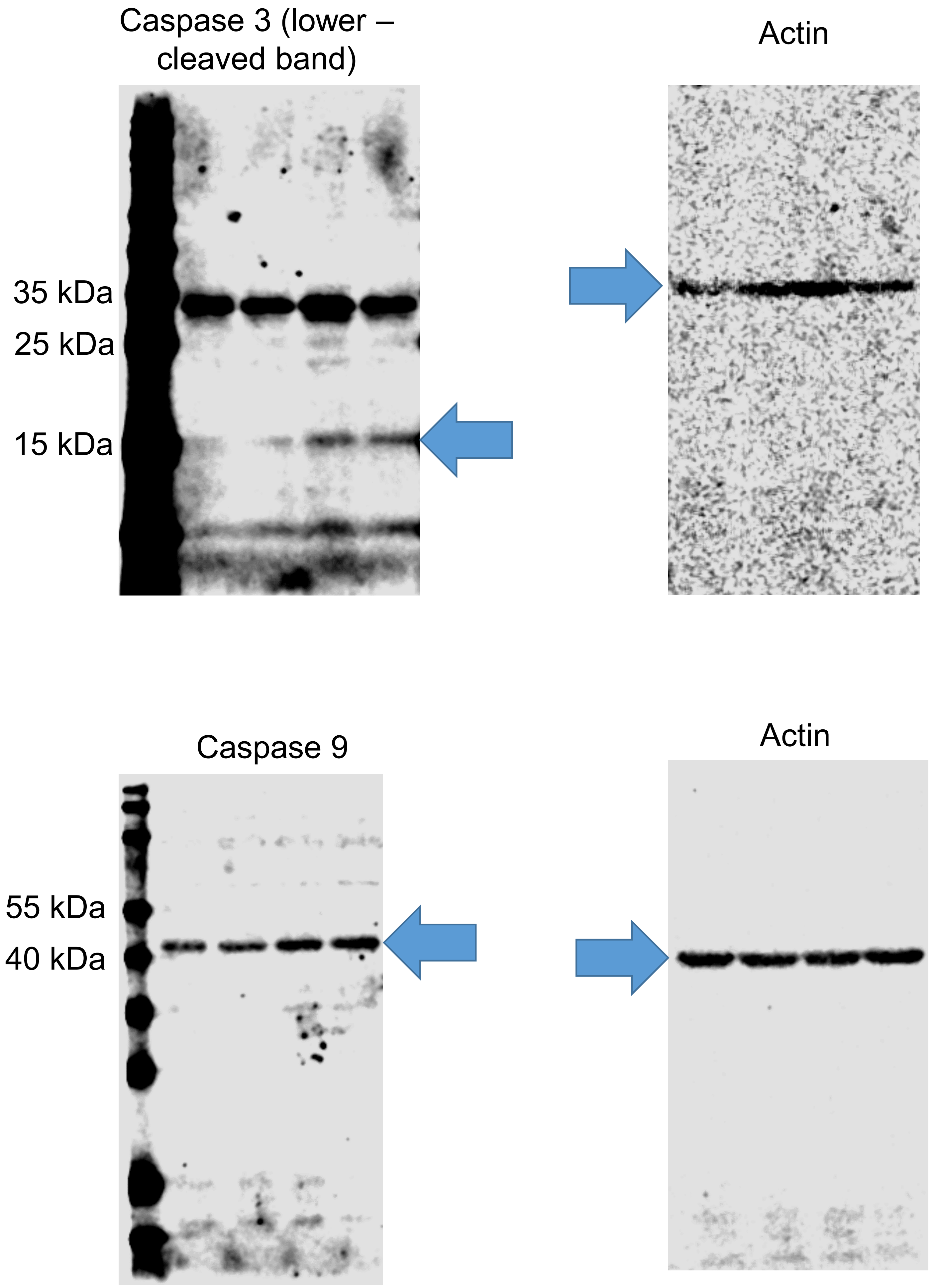
Caspase 3 (lower – cleaved band)
35 kDa
25 kDa
15 kDa
Actin
Caspase 9
55 kDa
40 kDa
Actin

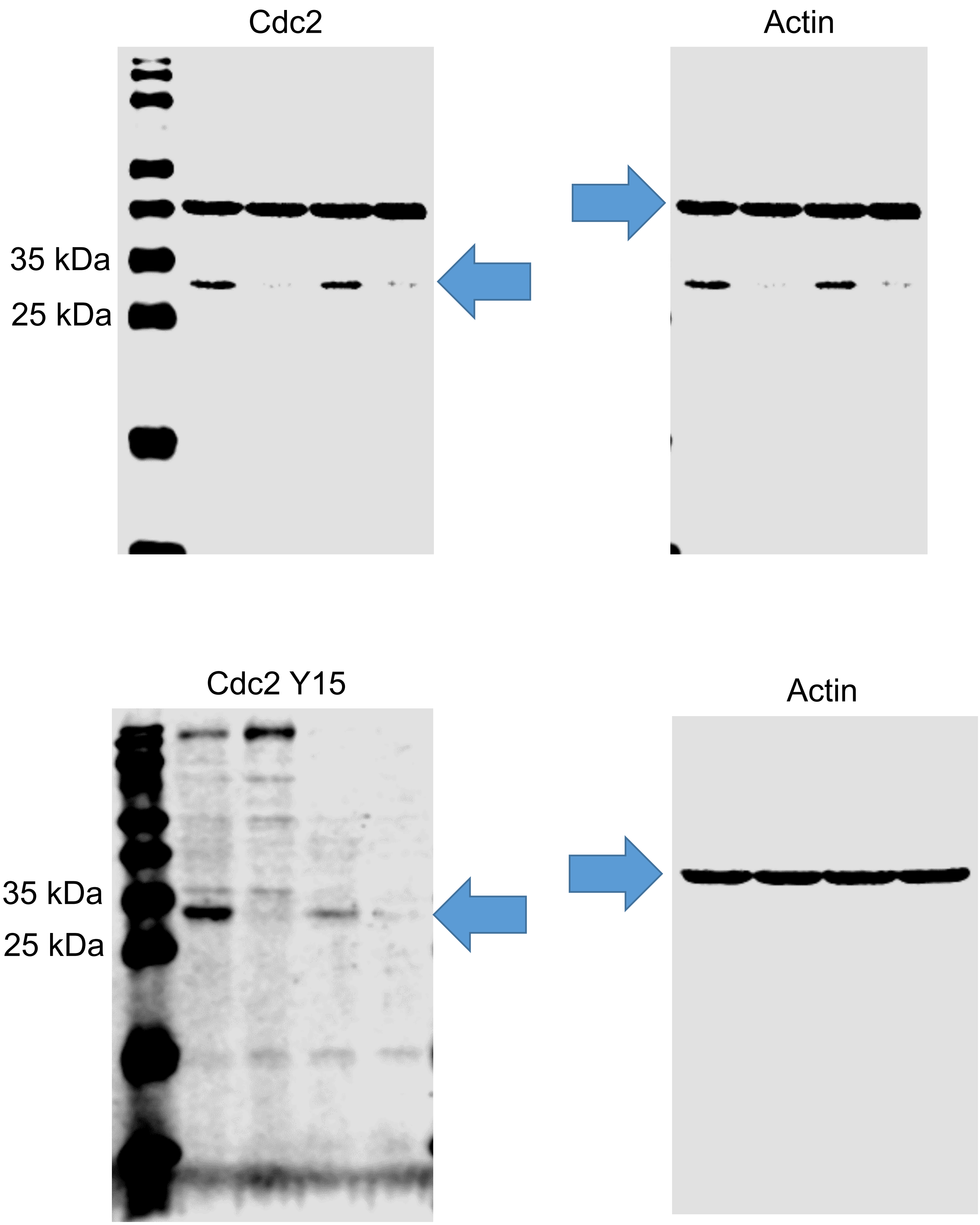

Cdc2
Actin
35 kDa
25 kDa
Cdc2 Y15
Actin
35 kDa
25 kDa

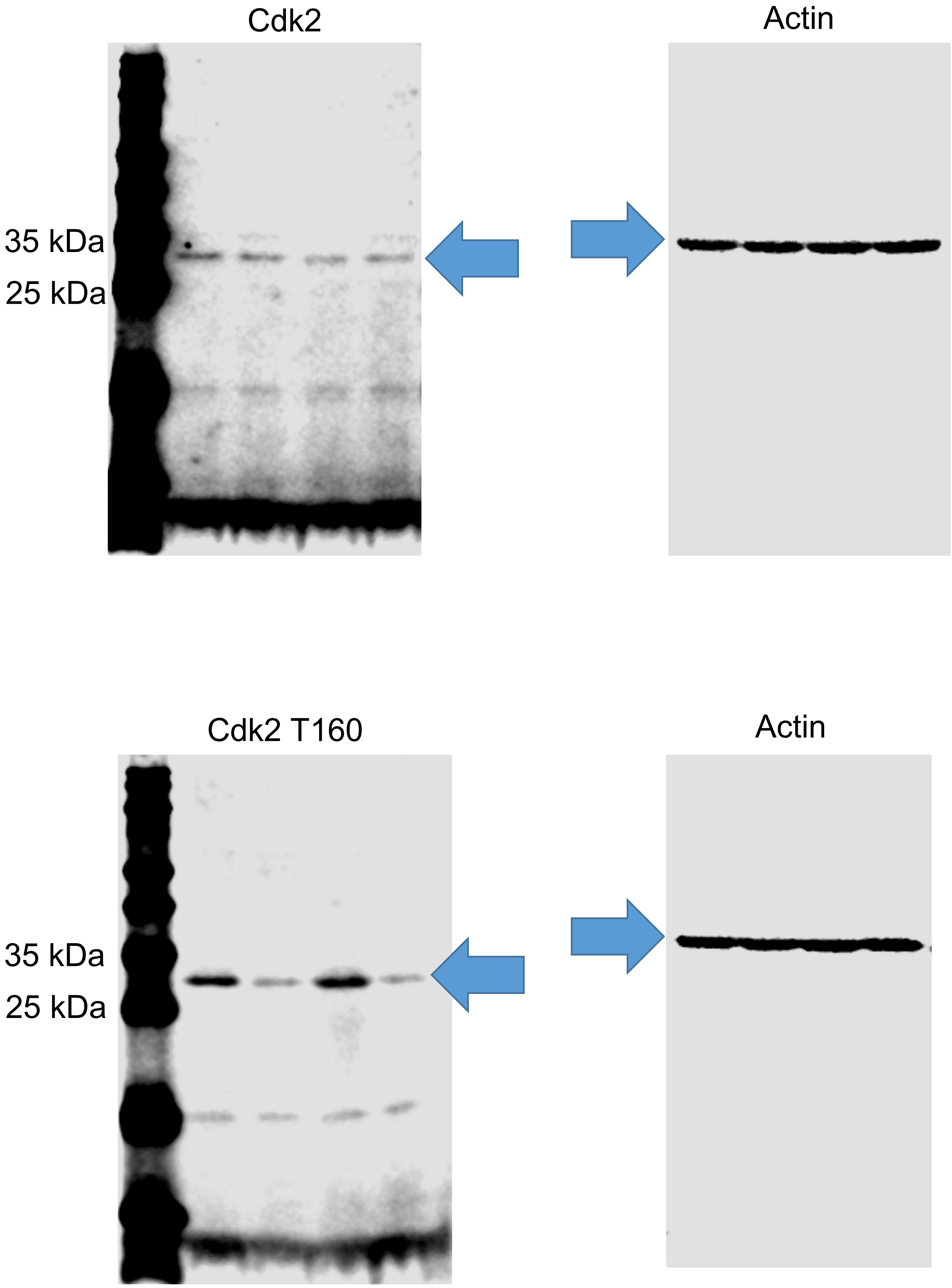
Cdk2
Actin
35 kDa
25 kDa
Cdk2 T160
Actin
35 kDa
25 kDa

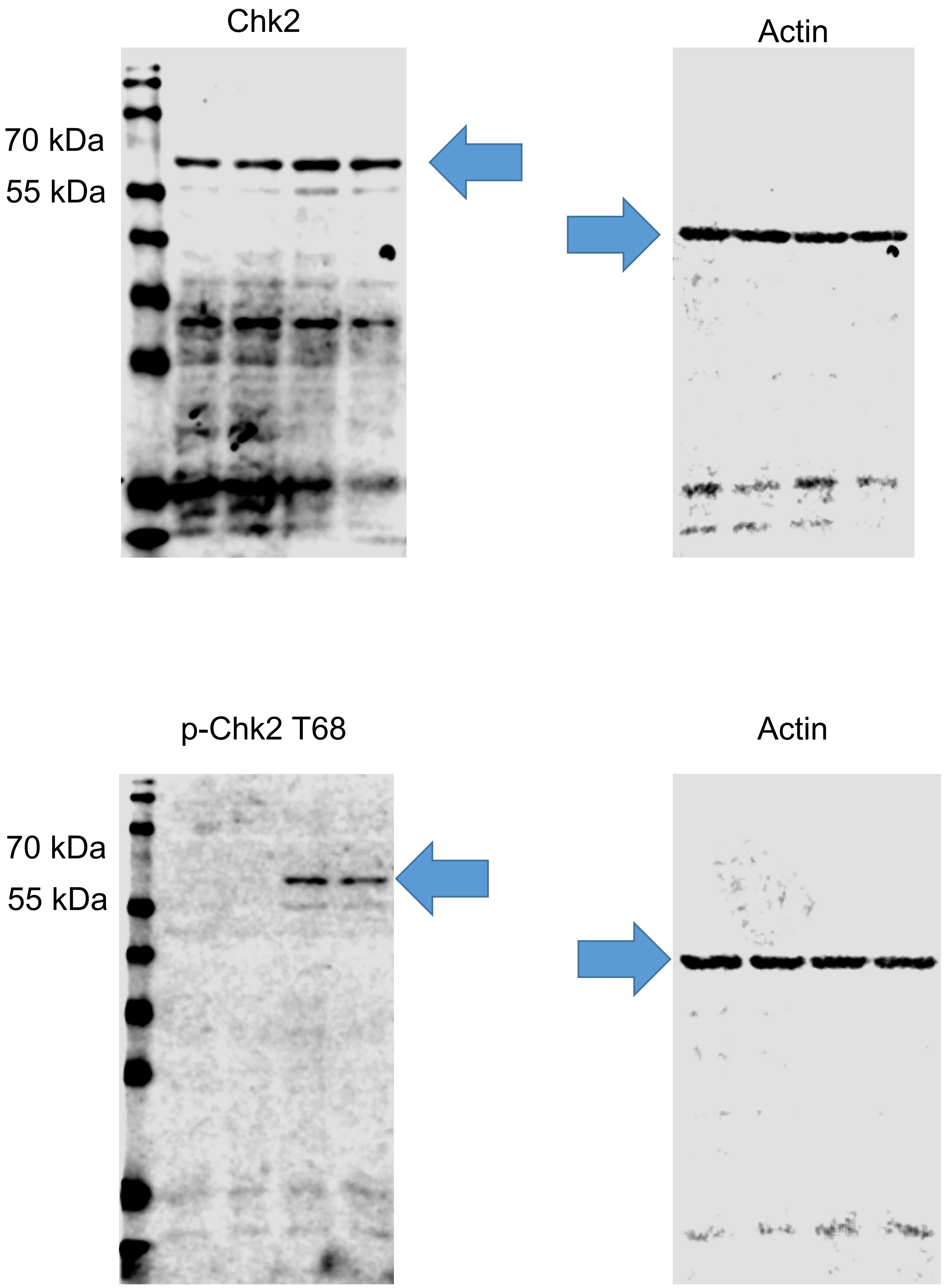
Chk2
Actin
70 kDa
55 kDa
p-Chk2 T68
Actin
70 kDa
55 kDa

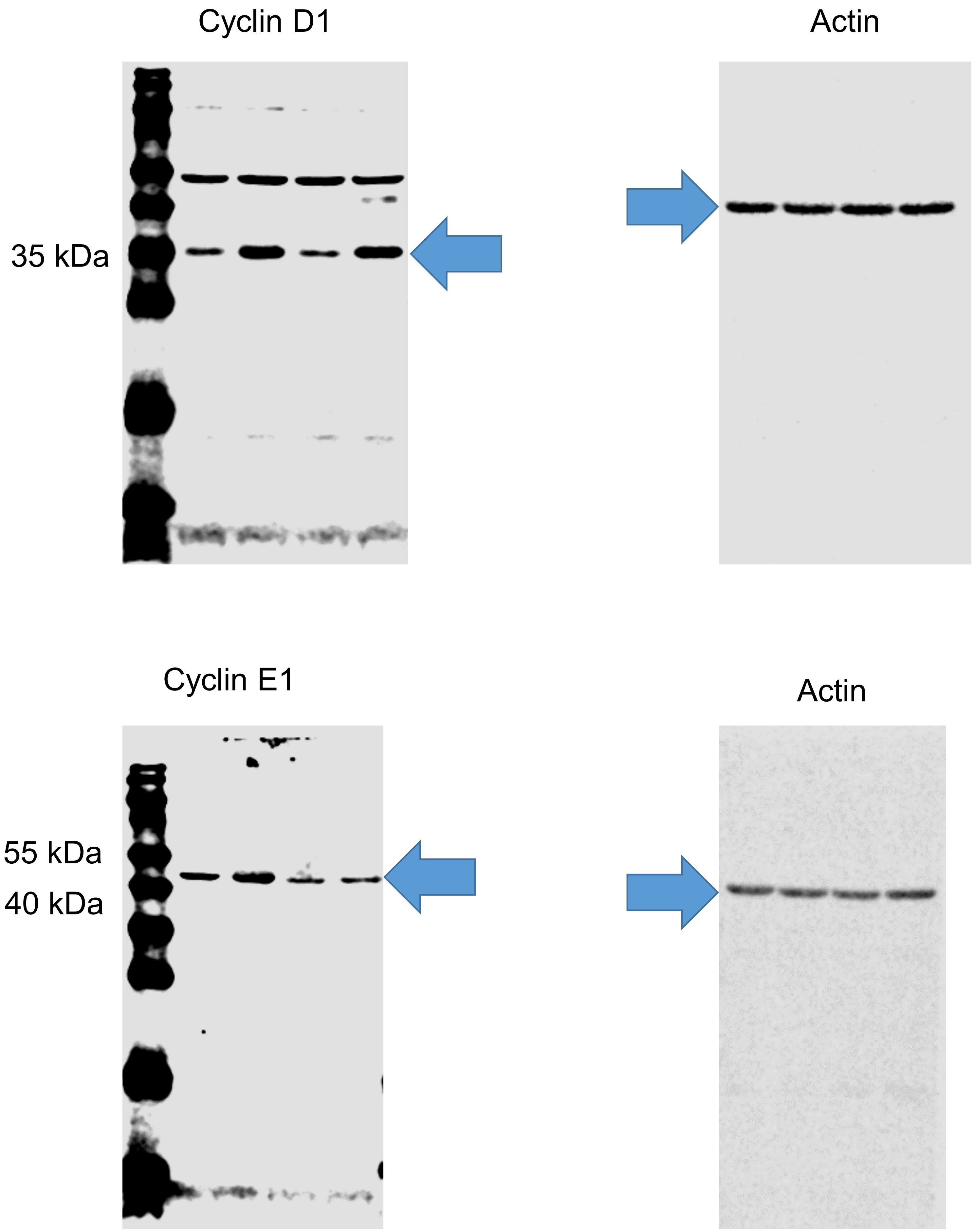
Cyclin D1
Actin
35 kDa
Cyclin E1
Actin
55 kDa
40 kDa

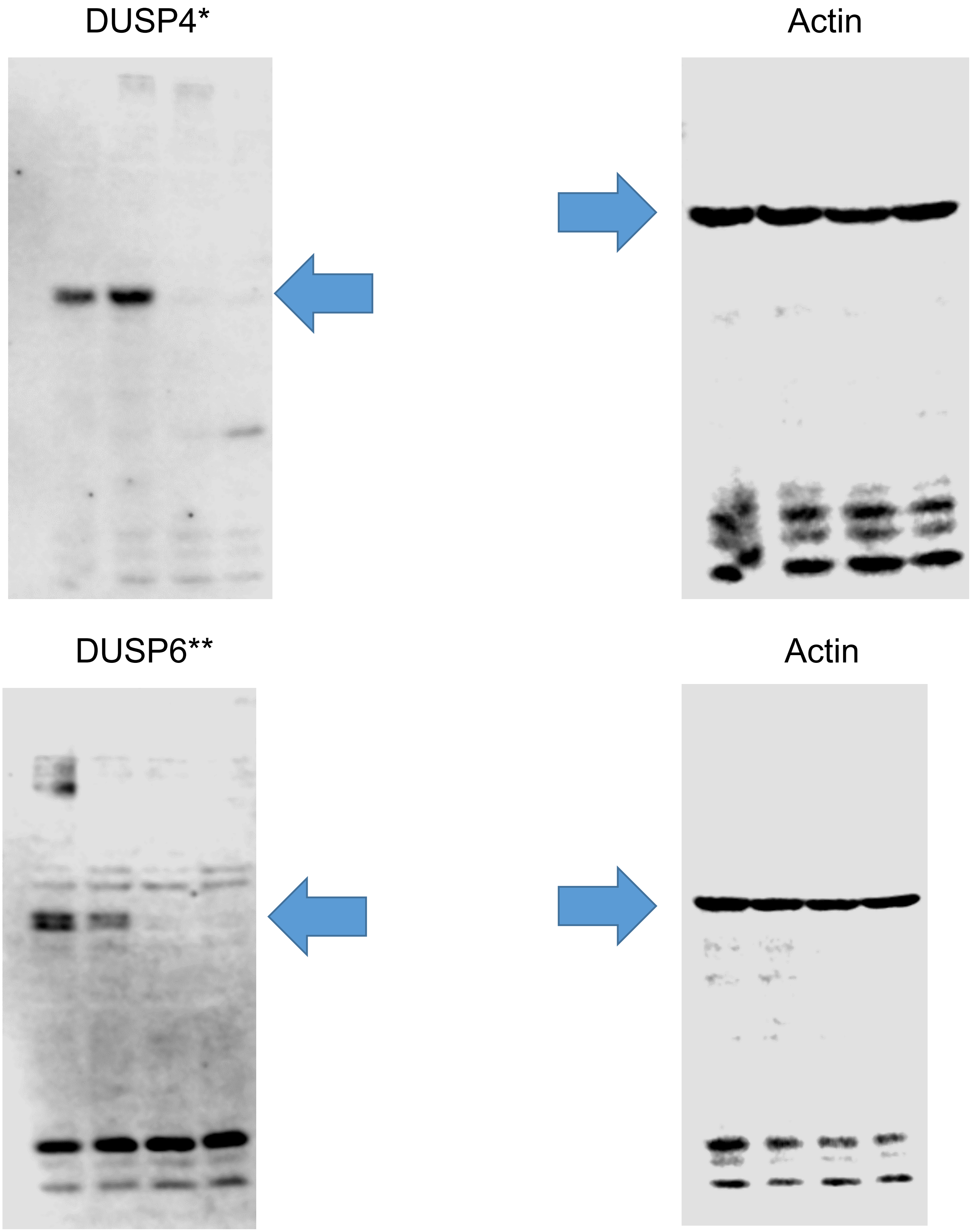

*MEK1/2, p-MEK1/2 (S217/221) and DUSP4 had the same molecular weight marker.
**p53, p-p53 S15 and DUSP6 had the same molecular weight marker.

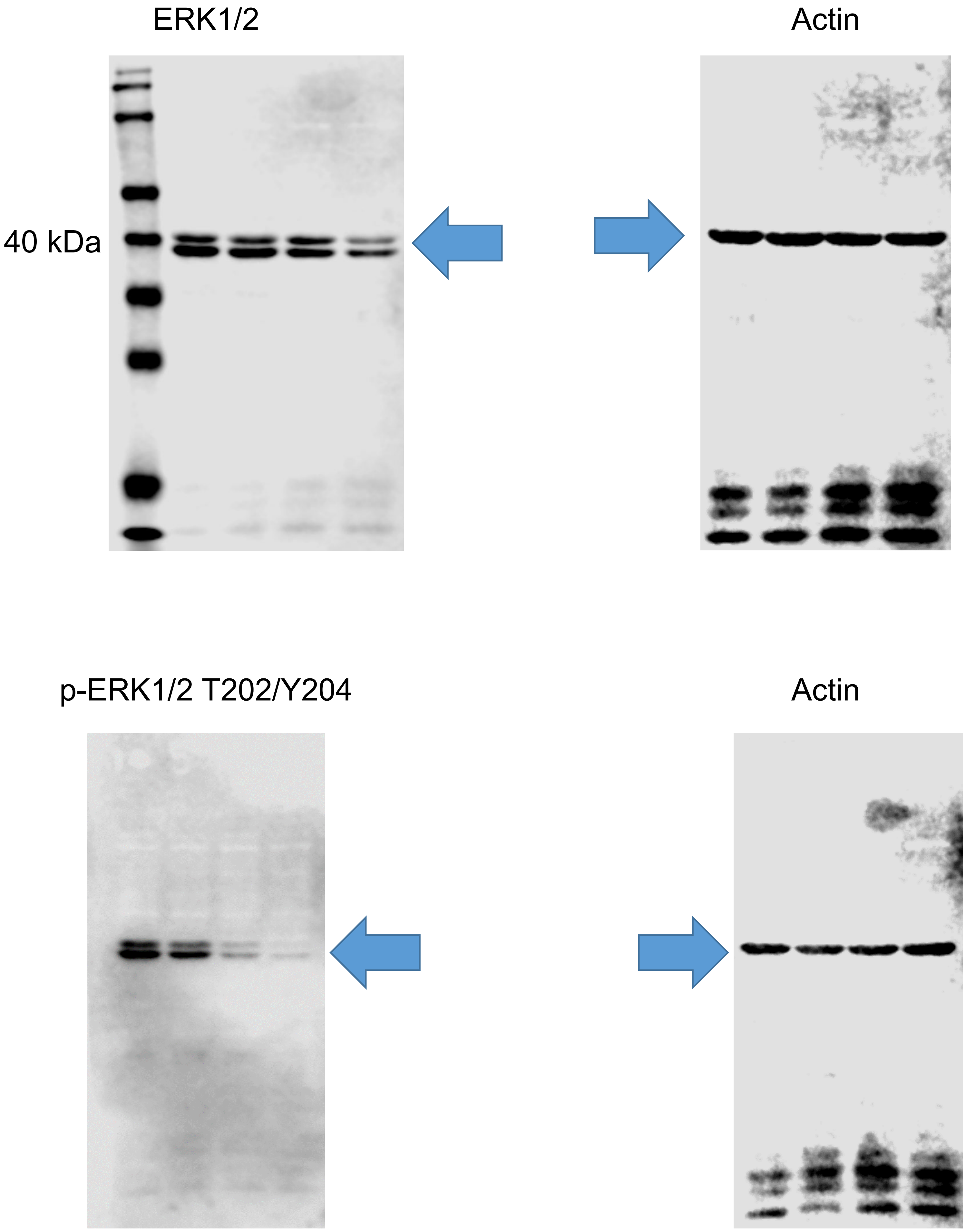
ERK1/2
Actin
40 kDa
p-ERK1/2 T202/Y204
Actin

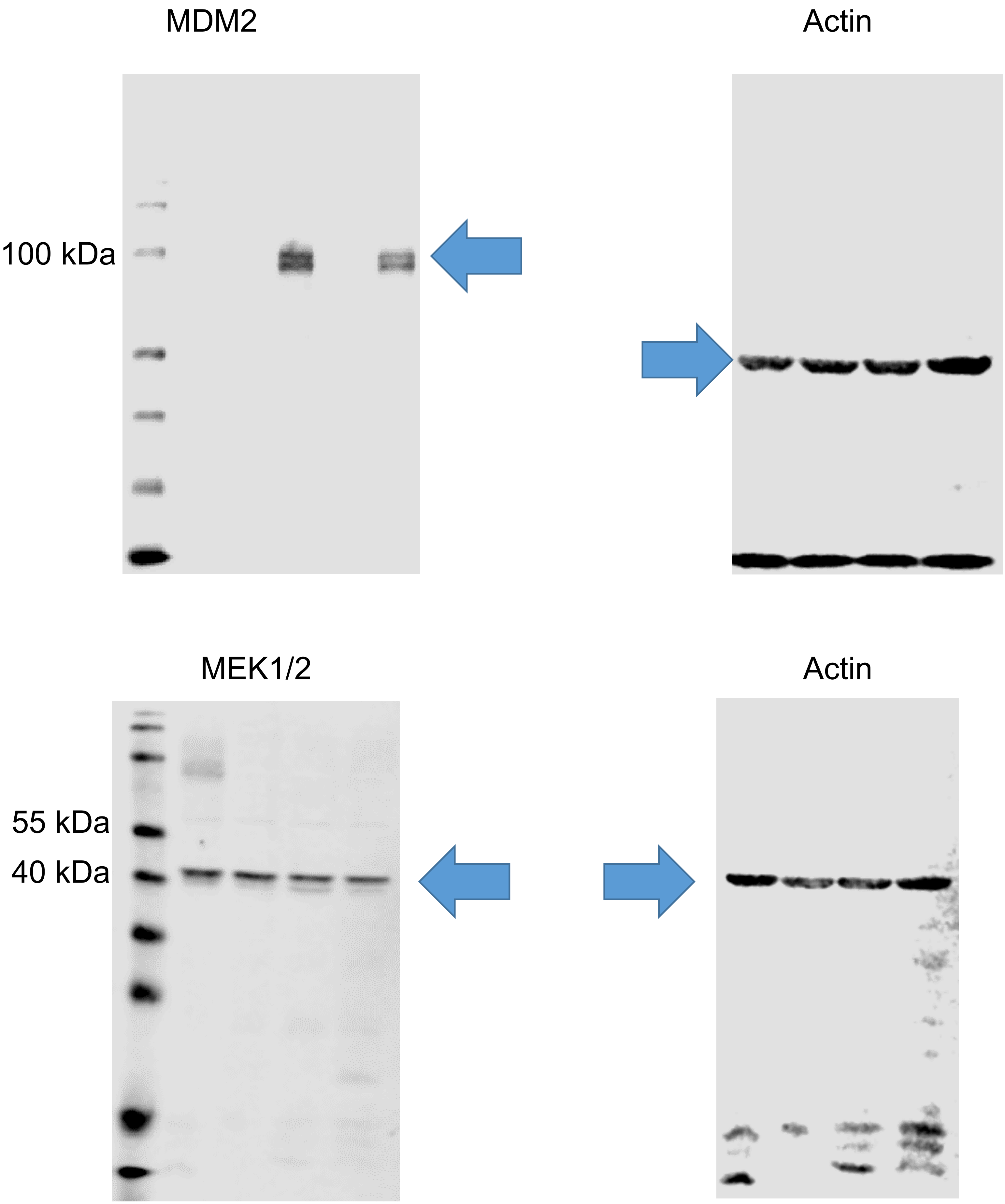
MDM2
Actin
100 kDa
MEK1/2
Actin
55 kDa
40 kDa

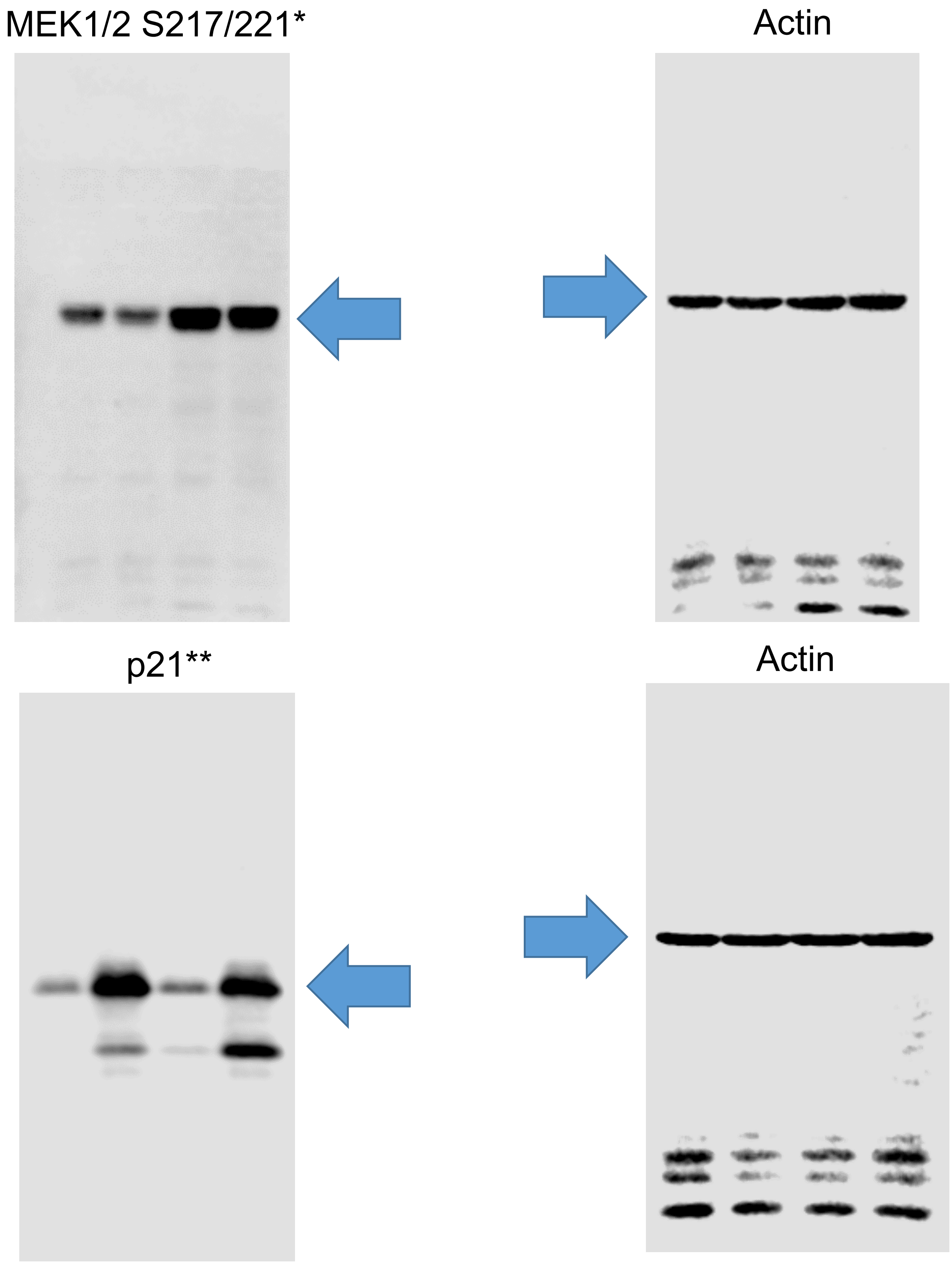

*MEK1/2, p-MEK1/2 (S217/221) and DUSP4 had the same molecular weight marker.
**BAX, p21 and PUMA had the same molecular weight marker.

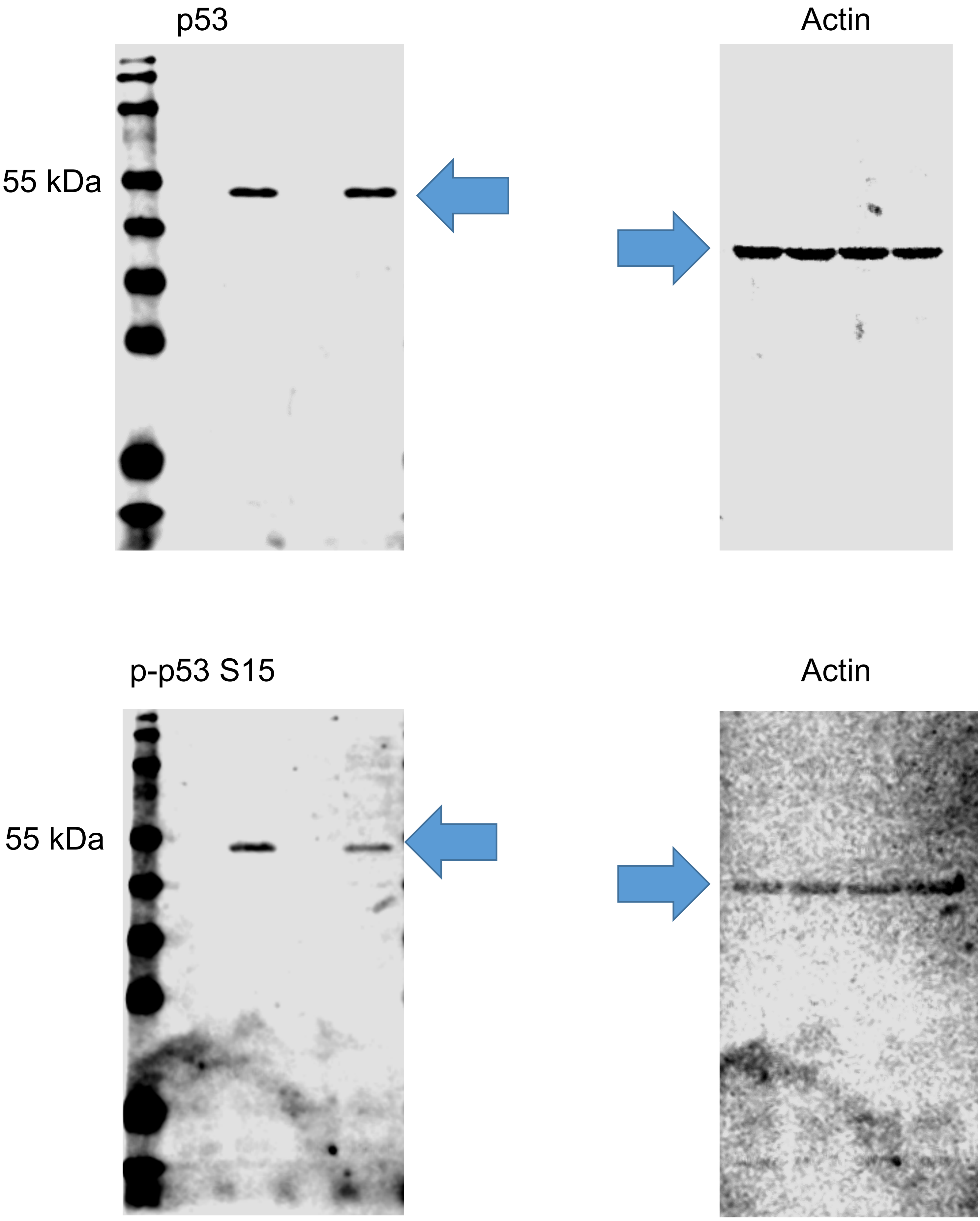
p53
Actin
55 kDa
p-p53 S15
Actin
55 kDa

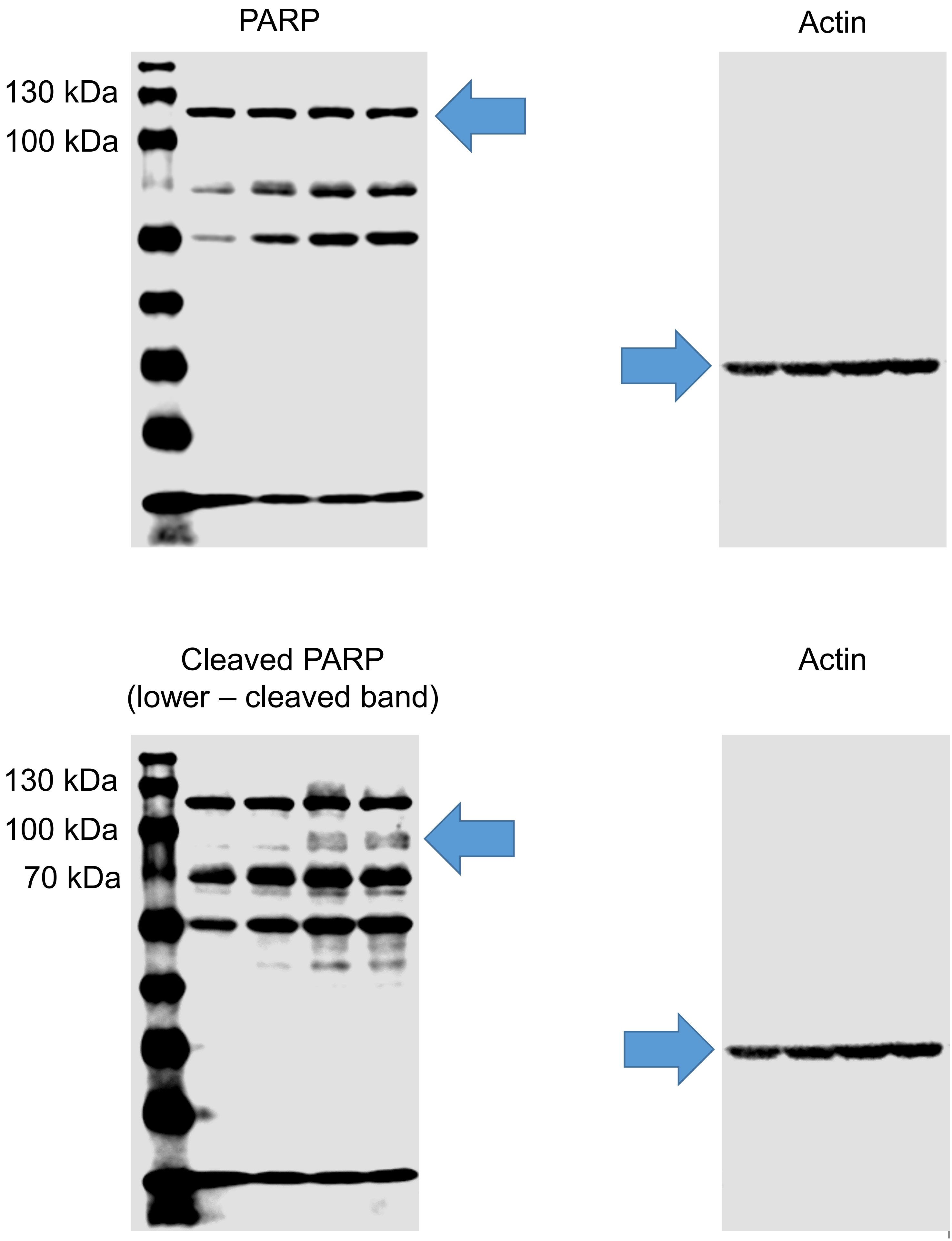
PARP
Actin
130 kDa
100 kDa
Cleaved PARP
(lower – cleaved band)
Actin
130 kDa
100 kDa
70 kDa

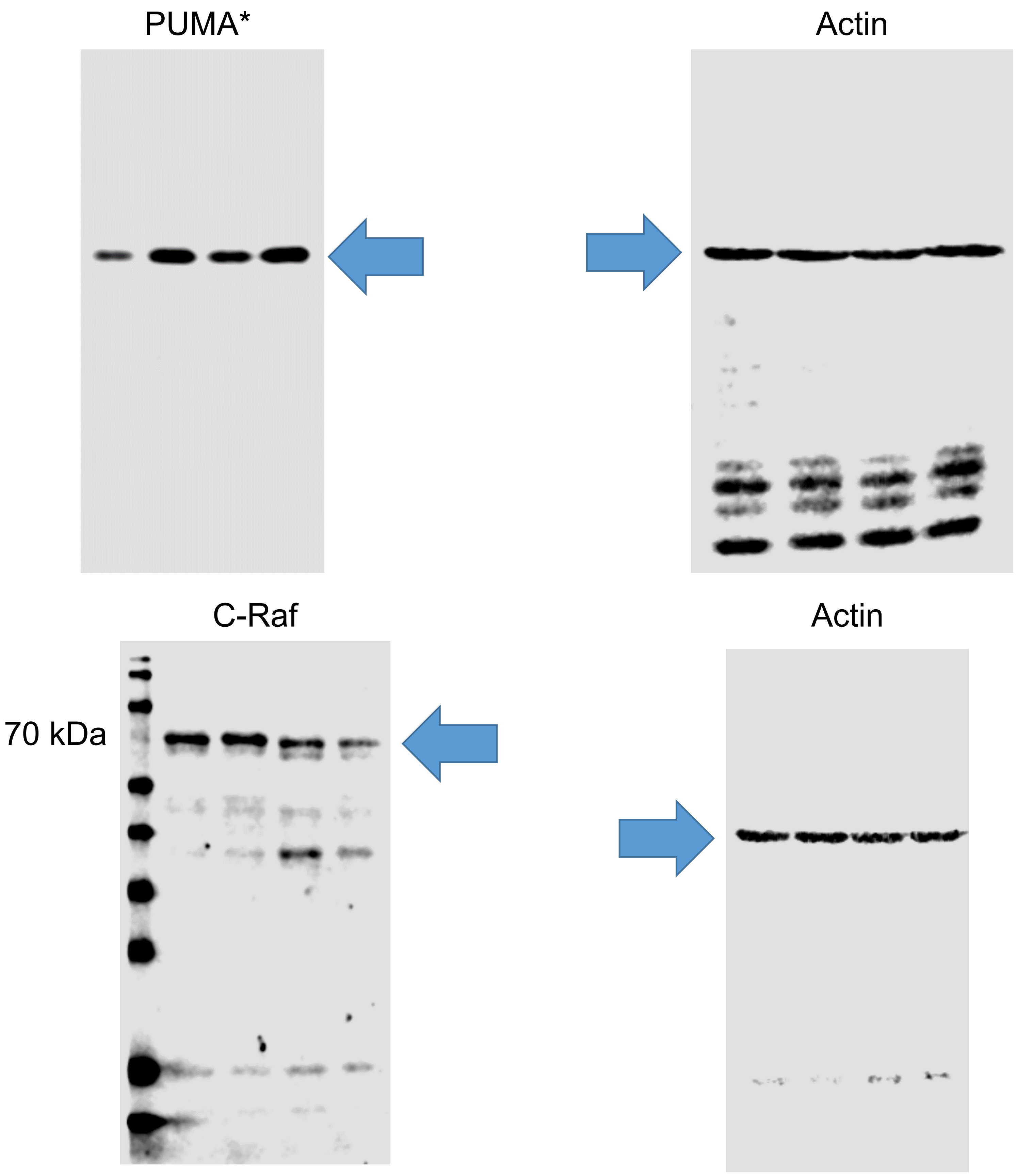

*BAX, p21 and PUMA had the same molecular weight marker.

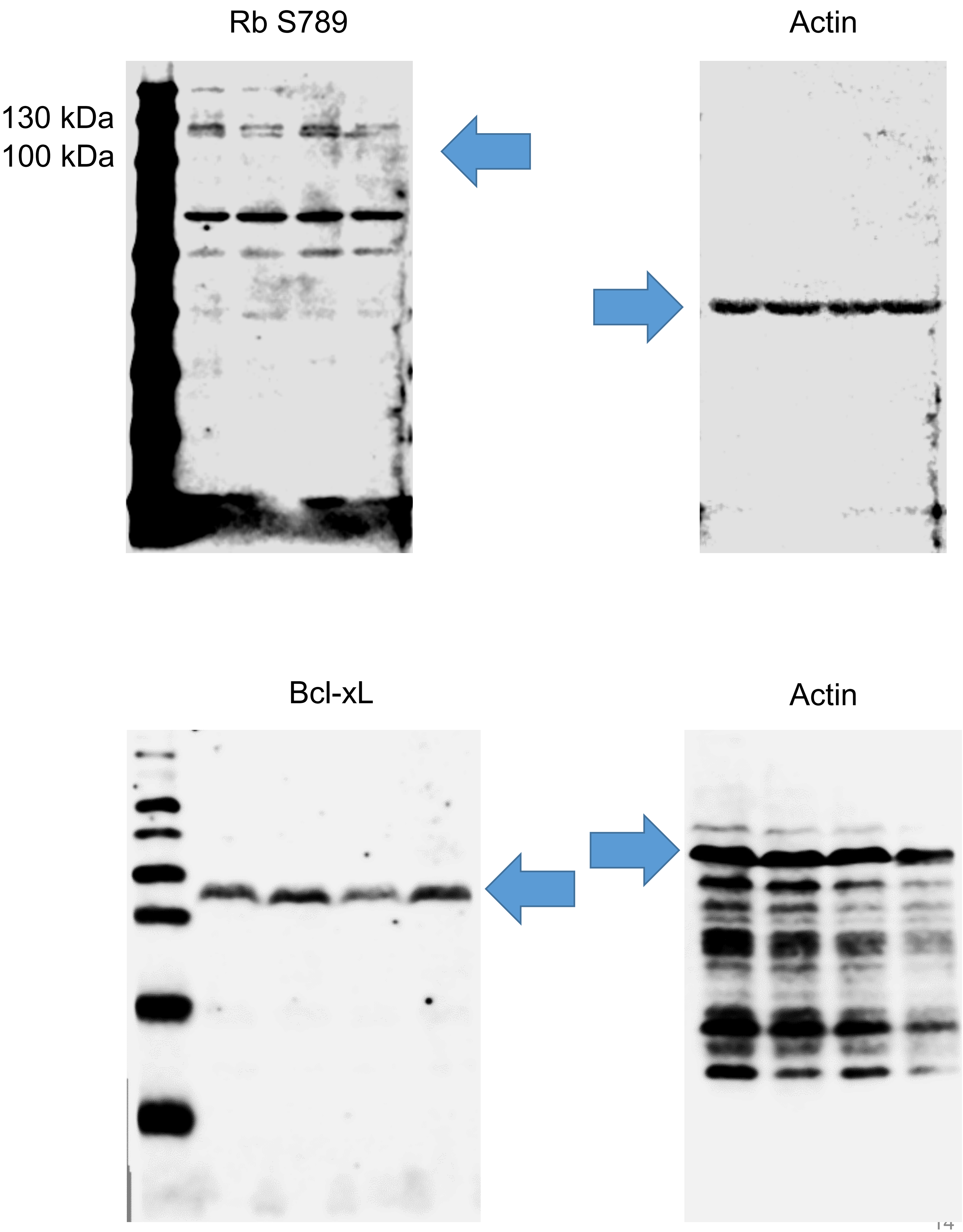
Rb S789
Actin
130 kDa
100 kDa
Bcl-xL
Actin

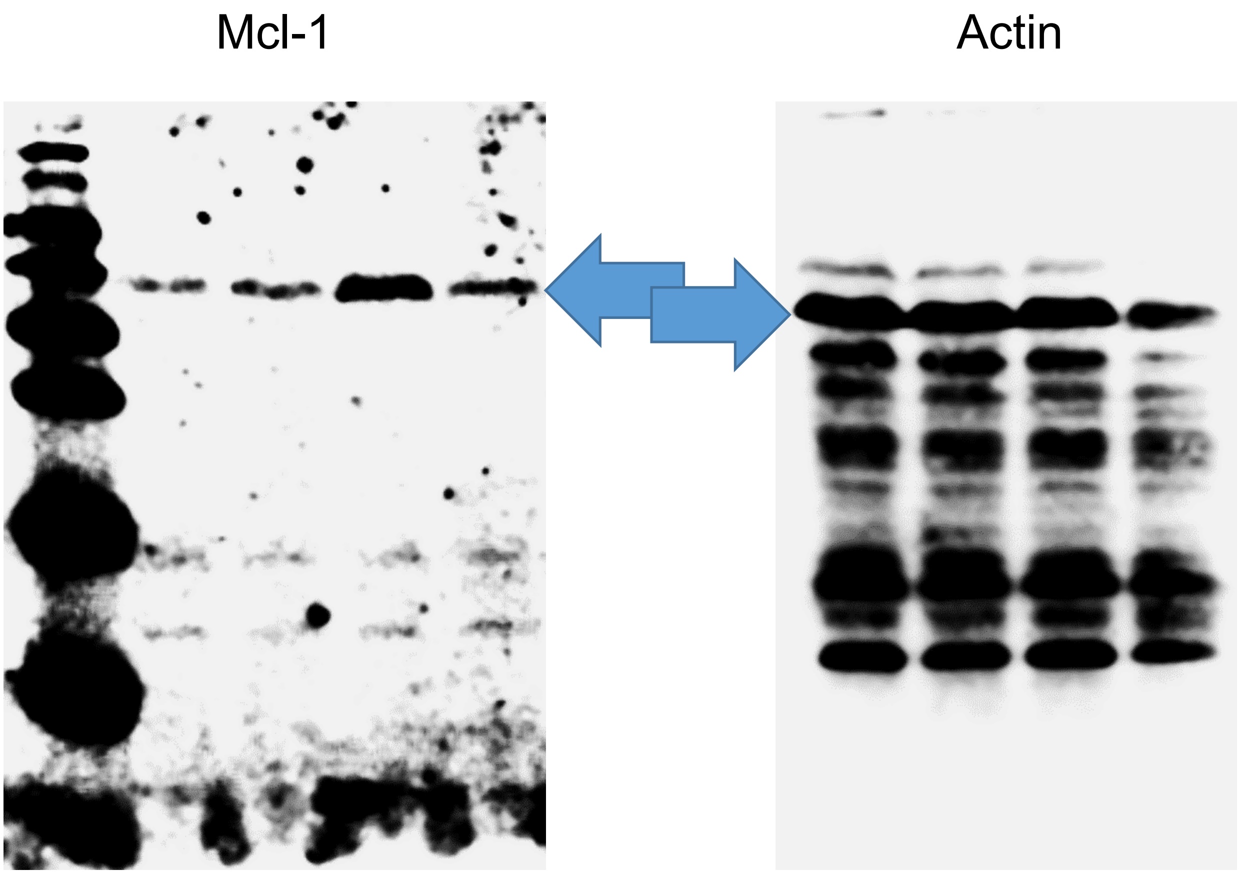
Mcl-1
Actin
